# Robust, Comprehensive Molecular, and Phenotypical Characterisation of Atypical *Candida albicans* Clinical Isolates From Bogotá, Colombia

**DOI:** 10.3389/fcimb.2020.571147

**Published:** 2020-12-02

**Authors:** Giovanni Rodríguez-Leguizamón, Andrés Ceballos-Garzón, Carlos F. Suárez, Manuel A. Patarroyo, Claudia M. Parra-Giraldo

**Affiliations:** ^1^Hospital Universitario Mayor Méderi-Universidad del Rosario, Bogotá, Colombia; ^2^School of Medicine and Health Sciences, Universidad del Rosario, Bogotá, Colombia; ^3^Unidad de Proteómica y Micosis Humanas, Grupo de Enfermedades Infecciosas, Departamento de Microbiología, Facultad de Ciencias, Pontificia Universidad Javeriana, Bogotá, Colombia; ^4^Biomathematics Department, Fundación Instituto de Inmunología de Colombia (FIDIC), Bogotá, Colombia; ^5^Molecular Biology and Immunology Department, Fundación Instituto de Inmunología de Colombia (FIDIC), Bogotá, Colombia

**Keywords:** *Candida albicans*, *Candida africana*, atypical isolates, pathogenicity, antifungal susceptibility, multilocus (MLST) genotypes

## Abstract

*Candida albicans* is commensal in human microbiota and is known to be the commonest opportunistic pathogen, having variable clinical outcomes that can lead to up to 60% mortality. Such wide clinical behaviour can be attributed to its phenotypical plasticity and high genetic diversity. This study characterised 10 Colombian clinical isolates which had already been identified as *C. albicans* by molecular tests; however, previous bioinformatics analysis of protein mass spectra and phenotypical characteristics has shown that this group of isolates has atypical behaviour, sharing characteristics of both *C. africana* and *C. albicans*. This study was aimed at evaluating atypical isolates’ pathogenic capability in the *Galleria mellonella* model; susceptibility profiles were determined and MLST was used for molecular characterisation. Cluster analysis, enabling unbiased bootstrap to classify the isolates and establish their cluster membership and e-BURST, was used for establishing clonal complexes (CC). Both approaches involved using representative MLST data from the 18 traditional *C. albicans* clades, as well as *C. albicans*-associated and minor species. Ten atypical isolates were distributed as follows: 6/10 (B71, B41, B60, R6, R41, and R282) were grouped into a statistically well-supported atypical cluster (AC) and constituted a differentiated CC 6; 2/10 of the isolates were clearly grouped in clade 1 and were concurrent in CC 4 (B80, B44). Another 2/10 atypical isolates were grouped in clade 10 and concurred in CC 7 (R425, R111); most atypical isolates were related to geographically distant isolates and some represented new ST. Isolates B41 and R41 in the AC had greater virulence. Isolate B44 was fluconazole-resistant and was grouped in clade 1. The atypical nature of the isolates studied here was demonstrated by the contrast between phenotypical traits (*C. africana*-like), molecular markers (*C. albicans*-like), virulence, and antifungal resistance, highlighting the widely described genetic plasticity for this genus. Our results showed that the atypical isolates forming well-differentiated groups belonged to *C. albicans*. Our findings could contribute towards developing molecular epidemiology approaches for managing hospital-acquired infection.

## Introduction

*Candida albicans* has been recognised as a member of healthy humans’ fungal microbiome (Zhang et al., 2017); however, it has been described that this opportunist fungi’s proliferation in suitable conditions can have a serious impact on its host’s health (this has led to it being defined recently as a pathobiont) (Sam et al., 2017). The range of pathologies associated with *C. albicans* proliferation includes localised oral and urogenital infections as well as cases of invasive fungal disease (IFD), having mortality percentages which can reach 60% (Mayer et al., 2013; Kadosh, 2019).

Concomitant diseases leading to the weakening of the immune system in intra-hospital (Costa-de-oliveira and Rodrigues, 2020) and immunosuppression populations favours the occurrence of clinical pictures associated with *C. albicans*, demonstrating the important role of a host’s immune state (Znaidi, 2020). However, this microorganism’s phenotypical plasticity has been identified during the last few years as the aspect contributing most to its successful proliferation, mainly regarding the expression of virulence factors, since this could provide it with the flexibility to survive in a target host’s hostile conditions and make it tolerant to treatment schemes (Basmaciyan et al., 2019).

*C. albicans* detection and monitoring strategies have been based on descriptions of clinical isolates’ phenotypical and microbiological characteristics, taking the species’ ability to grow at 42°C, shorter germ tube formation, inability to produce chlamydospores and assimilate trehalose and/or amino-sugars as indicators (Tietz et al., 1995).

Such assays have contributed towards clarifying the epidemiological panorama in some regions worldwide (Borman et al., 2013; Chowdhary et al., 2017); however, disagreement amongst regarding these assays’ results and the strains’ clinical impact have revealed the need for introducing more robust assays/tests for describing the isolates. This is why proteomics and molecular profile-based tests have recently gained importance for more precisely detecting *C. albicans* infection events (Chowdhary et al., 2017).

Our research group adopted a strategy in 2007 for describing the local epidemiology of *C. albicans* and that of related *Candida* species; this study classified 101 clinical *Candida* isolates obtained from 10 tertiary care hospitals in Bogotá, Colombia, using microbiological tests. MALDI-TOF-MS confirmed 31 of them as *C. albicans* (Rodríguez-Leguizamón et al., 2015b). Such screening revealed the importance of the intra-hospital spread of *C. albicans* in Colombia and led to describing circulating strains’ antifungal susceptibility profiles (Rodríguez-Leguizamón et al., 2015a).

Incongruity has been identified between traditional assays and proteomic and molecular strategies (using individual genes) (Alonso-vargas et al., 2008), demonstrating atypical clinical isolates in Spain (*C. dubliniensis*) (Albaina et al., 2015) and Colombia (*C. albicans*) (Rodríguez-Leguizamón et al., 2015b). This identified a set of isolates having phenotypical characteristics typical of *C. africana*, identified as *C. albicans/africana* by MALDI-TOF MS. However, molecular tests (D1/D2 rRNA and HWP1) and direct analysis of MALDI-TOF spectra showed that these atypical isolates were related to *C. albicans* (Rodríguez-Leguizamón et al., 2015b).

This required *C. albicans* typing strategies having greater discrimination power, such as the multilocus sequence typing (MLST)-based strategy which is extremely useful for identifying the types of sequences circulating in different regions of the world (Scordino et al., 2018). It currently has an easy access, debugged database, involving low complexity analysis (Pérez-Losada et al., 2013). A proposal regarding population structure for *C. albicans* based on 18 main clades agreed with epidemiological characteristics, such as the infection’s anatomical location, geographical distribution, and susceptibility to antifungal drugs (Bougnoux et al., 2004; McManus and Coleman, 2014); however, such associations are not absolute and have become even more diversified with an increase in the isolates making up the baseline.

Considering the incongruities regarding assay results when using the set of Colombian atypical *C. albicans* isolates and completing their phenotypical characterisation involved pathogenicity assays concerning antifungal susceptibility profiles in the *Galleria mellonella* model. MLST was used for determining diploid sequence types (DSTs) for these atypical isolates; this led to obtaining information for hierarchical cluster analysis and defining related DST groups (using the BURST algorithm) for describing these isolates’ relationships with representative strains from the 18 clades traditionally accepted for *C. albicans* and from other *C. albicans*-related species (*C. africana* and *C. dubliniensis*).

## Methods

### Ethics Statement

Both the Universidad del Rosario and Hospital San Ignacio ethics’ committees (i.e. its associated institution) approved this study.

### Strains and Isolates

Ten atypical clinical isolates have already been reported by our group. These strains were collected from third-level hospitals in Bogotá, Colombia, and then characterised by phenotypic and MALDI-TOF MS using the Bruker Daltonics protocol (Rodríguez-Leguizamón et al., 2015b).

### DNA Extraction and Molecular Characterisation

The genomic DNA (gDNA) used in this study was extracted from a pellet of isolates grown on Sabouraud agar (in previously described incubation conditions), using an UltraClean Microbial DNA isolation kit (Mo Bio Laboratories, Solana), following the manufacturer’s instructions. The DNA used for the isolates’ molecular characterisation involved two procedures: amplifying the HWP1 gene (for discriminating between species) (Romeo and Criseo, 2008; Hazirolan et al., 2017) and amplifying and sequencing the seven housekeeping genes in the *C. albicans-*standardised MLST scheme (Bougnoux et al., 2003). Both approaches involved adding 50 ng DNA to a 25 μl volume for the PCR reaction, using a Kapa HiFi PCR kit (KAPA Biosystems). These genes were amplified using 5 min cycles at 94°C for primary denaturing, followed by 35 cycles at 94°C (30s), 55°C (60 s), and 72°C (60 s), with a final 5 min extension step at 72°C. The amplicons were visualised by electrophoresis on 1.5% agarose gels, stained with SYBR Safe DNA Gel Stain (Invitrogen). The sequenced products had already been purified using a Wizard SV Gel and PCR Clean-Up System (Promega) and both strands were subsequently sequenced by the dideoxy terminal method (Sanger sequencing) by Macrogen (Korea).

### Cluster Analysis Using MLST Data

**Figure 1:**
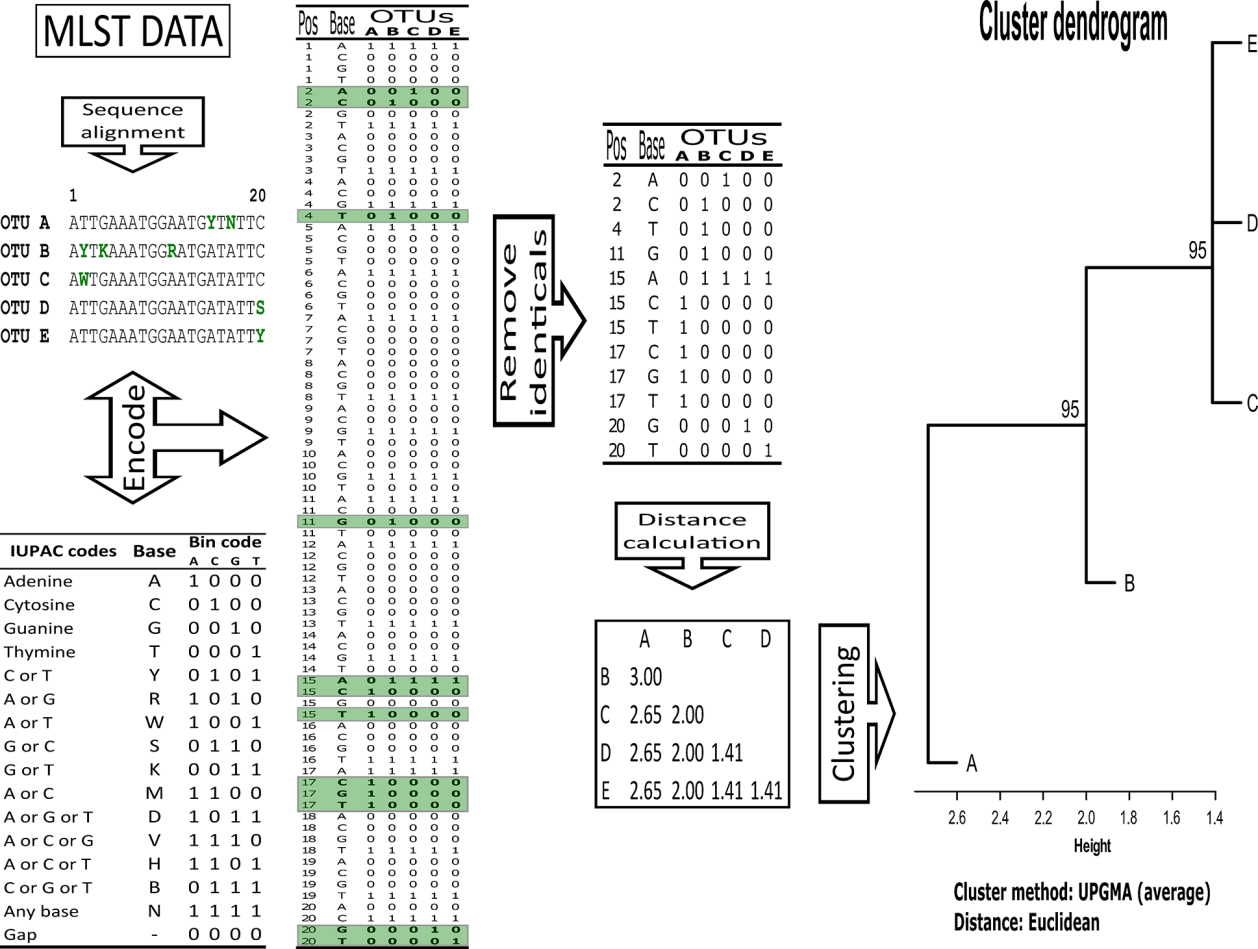
Overview of the methodological pipeline for MLST data cluster analysis.

Together with the atypical Colombian isolates (10 isolates), a representative set of MLST data available for *C. albicans* (clades 1 to 18, 82 isolates), two for *C. africana* isolates and one for *C. dubliniensis* was analysed (95 isolates in total) (**Table 1**). Clustal Omega was used for aligning the gene sequences (Sievers et al., 2011) which were then encoded using four binary digits for each IUPAC nucleic acid notation symbol and all identical positions were removed from the resulting binary matrix. Cluster analysis was performed for each marker and all were concatenated using Euclidean metrics and the unweighted pair group method with arithmetic mean (UPGMA). Unbiased bootstrap values were used to support the trees (10,000 replicas) using the pvclust package in R 3.4.3 (Suzuki and Shimodaira, 2006; Dean and Nielsen, 2007). A cluster having a ≥95 bootstrap value was considered supported (**Figure 1**).

**Table 1:** The isolates analysed here, including origin, source, country, ST numbers, and CC.

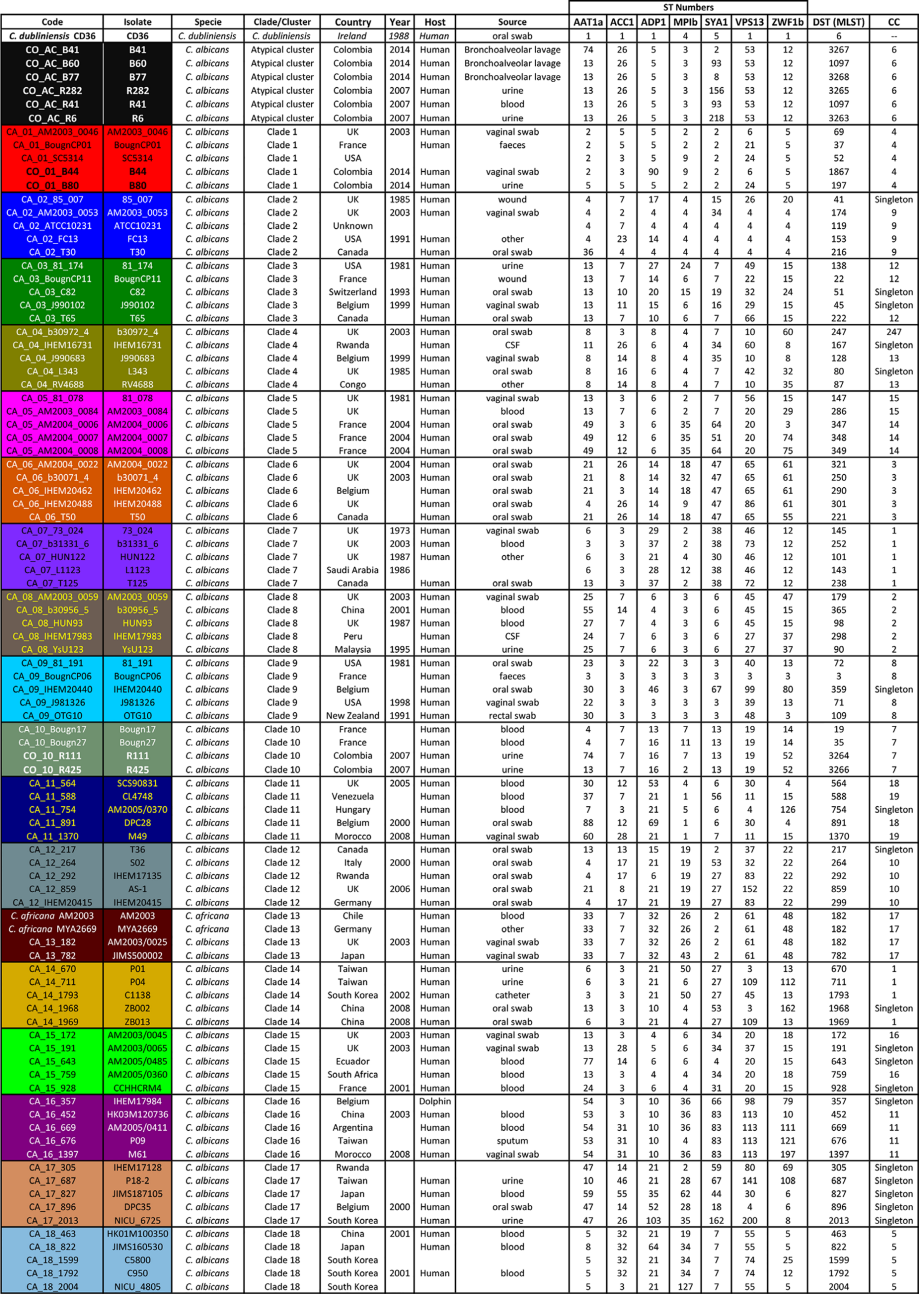	

Each isolate’s membership in a particular cluster was evaluated by separately analysing its clustering pattern for each gene. Membership was defined in terms of clade differentiation and neighbourhood. For example, a membership of 1 occurred if an isolate was in a clearly differentiated clade; a membership of 0.5 was assigned for each clade if it occurred in a group where two clades concurred, and so on. An isolate’s stability regarding MLST (7 genes: *AAT1a, ACC1, ADP1, MPIb, SYA1, VPS13*, and *ZWF1b*) was computed as the percentage at which a given isolate was observed in a clade (100% if it always occurred in a specific clade, or 14% if an isolate belonging to a clade occurred in one gene out of the 7 analysed here).

### BURST Analysis

The allele profiles from the set of data used for phylogenetic analysis (**Table 1**) were analysed for identifying closely related, delimited and mutually exclusive groups, defined as clone complexes (CC); the eBURST V3 package (http://eburst.mlst.net/) was used for this (Feil et al., 2004), considering triple-locus variation (TLV) as cut-off for delimiting groups. The goeBURST algorithm was used for creating minimum spanning trees providing information about evolutionary patterns in conditions comparable to those for most natural microbial populations (Francisco et al., 2009). STs having six or more different alleles were defined as singletons.

### Killing Assays in *G. mellonella*

Killing assays were performed in *G. mellonella*, as previously described (Cotter et al., 2000; Fuchs et al., 2010b; Fuchs et al., 2010a). Briefly, final (sixth) instar larvae weighing approximately 300 mg were used. Suspensions of individual *Candida* isolates which had been grown on Sabouraud agar for 24 h at 37°C were harvested by gently scraping colony surfaces with sterile plastic loops, washed twice in sterile phosphate-buffered saline (PBS), counted in haemocytometers and adjusted to 10^7^ cells/ml in sterile PBS. Individual larvae were inoculated with 10^5^ yeast (10 µl final inoculum volume) in the left rear proleg using a 0.5 ml BD syringe. At least ten larvae were inoculated per isolate per experiment (experiments involved using three independent isolates from each *Candida* test species). The larvae were monitored for 10 days and survival outcome was determined; larvae were considered dead when no response was observed following touch. Larval control groups received 10 µl sterile PBS in the same manner. Inoculated larvae were incubated at 37°C and scored for viability at 24 h intervals.

### Antifungal Susceptibility

Yeast isolates were tested for *in vitro* susceptibility by the agar diffusion method using Etest reagent strips for echinocandin (caspofungin - CAS), triazole (fluconazole - FLU), and polyene (amphotericin B - AMB), according to the manufacturer’s instructions (bioMérieux SA). Roswell Park Memorial Institute (RPMI) agar supplemented with 2% glucose was used as test medium for the assays. The 106 cell/ml yeast suspensions were spread uniformly on RPMI agar plates with sterile swabs and allowed to dry for 15 min. MIC readings for all agents were made following 24 h incubation at 35°C. MIC values were determined at the point of inhibition growth ellipse intersection with E-test strip. The MIC was read as the drug concentration that leads to complete inhibition 100% for amphotericin B and 80% inhibition for azoles and echinocandins. The MICs for *C. parapsilosis* ATCC 22019 and *C. krusei* ATCC 6258 quality control strains all came within reference ranges (data not shown).

### Statistical Analysis

All experiments involved using three independent biological replicates; GraphPad Prism 7.0. was used for creating survival curves following the Kaplan-Meier method. Yeast isolate cumulative survival was estimated, along with the mean ± standard deviations, medians and quartiles; overall and between-pair survival distributions were compared by Log Rank (Mantel-Cox) test with multiple Bonferroni comparison adjustment, using {∝* = 1- (1-∝) ^ [1/(# comparisons)]} significance level. Asymptotic likelihood ratio and Cox proportional hazards tests were used for comparing all isolates’ mortality rates after 10 days (a regression model was used for comparing proportional hazard ratios). Schoenfeld’s residue-based test had been used for checking the proportional hazards (PH) assumption. Statistical tests were evaluated at a 5% significance level of (with Bonferroni adjustment *p <*0.00465), using the statistical package SPSS 27.

## Results

### Cluster Analysis

**Figure 2:**
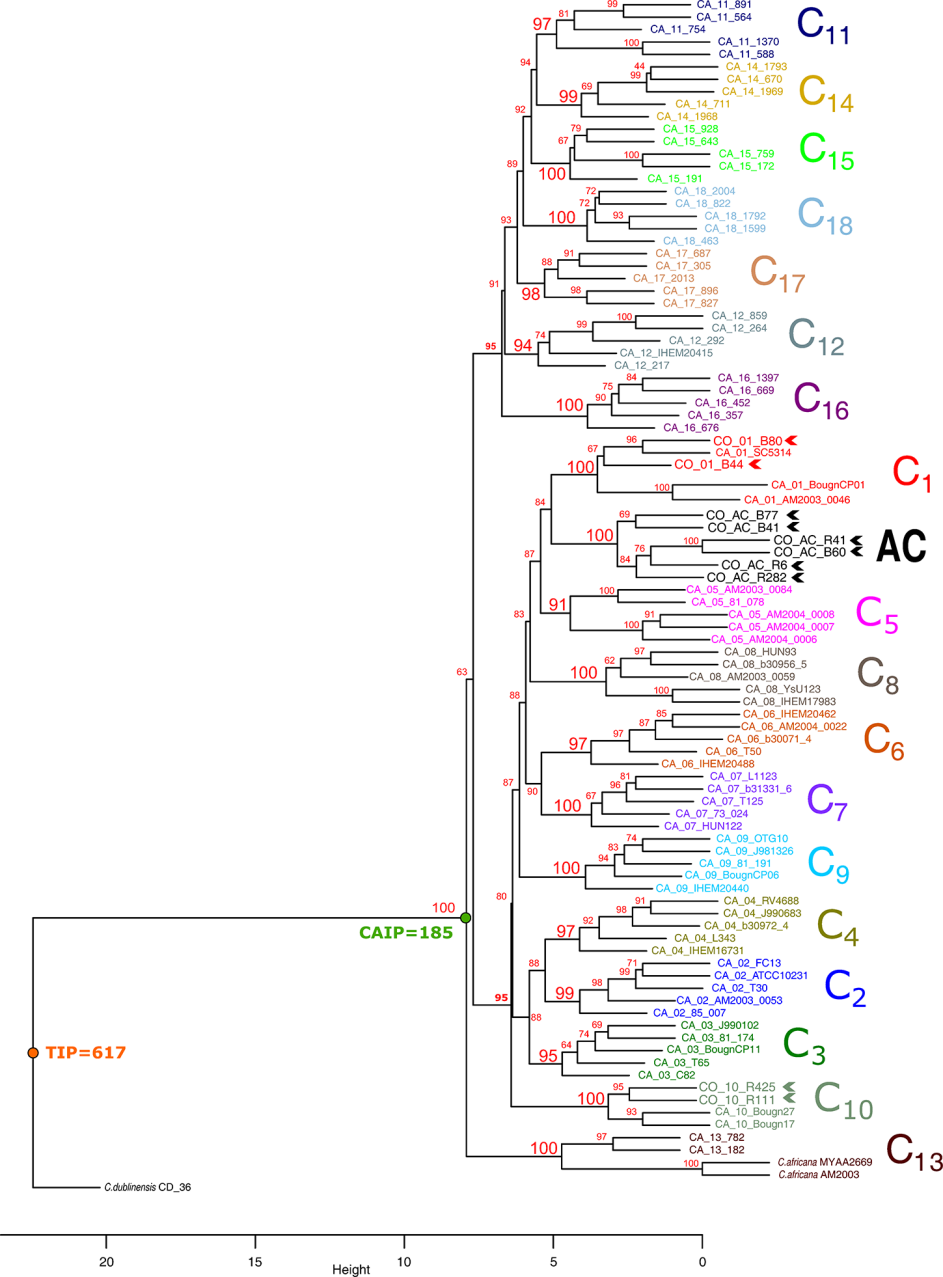
Cluster analysis of MLST data, using the UPGMA method. Each clade is shown in a different colour. Colombian isolates are indicated in bold and chevrons. Bootstrap values above 95% were considered significant. Large numbers in red denote support for each clade. TIP: total informative positions, CAIP: *C. albicans* informative positions.

The proposed pipeline (**Figure 1**) enabled constructing dendrograms in which Colombian isolates were assigned to the reported clades. MLST analysis (using the seven concatenated genes) differentiated all 18 C*. albicans* clades (**Figure 2**) and found 16/18 clades in well-supported clusters (clades 5 and 12 were nearly supported, having higher than 90% bootstrap values).

*C. albicans* clades were divided into two large groups; the first contained seven clades (11, 12, 14–18) and the second 10 (clades 1–10). Clade 13, having two *C. africana* and two *C. albicans* isolates, appeared as a basal branch, followed by *C. dubliniensis* isolate as outgroup. Colombian atypical isolates were grouped into clades 1 (2 isolates) and 10 (2 isolates). A well-supported and differentiated cluster could also be observed (named atypical cluster - AC: six isolates) which was associated with clade 1 (**Figure 2**).

#### *Candida albicans* Isolates Had Multiple Clade Memberships, Depending on the Locus Being Considered

Separate analysis of each gene for studying isolate membership of the different clades showed that relationships between clades varied significantly (**Table 2** and **Supplementary Figures** and **Tables A to G**). The total amount of informative positions (TIP) and the amount of informative positions just for *C. albicans* sequences (CAIP) were reduced by analysing each locus separately. The informative positions defining *C. albicans* clade classification varied between 36 (for SYA1) and 13 (ACC1). Given the reduction in the amount of informative positions considered, it was to be expected that differentiation support and capacity for the clusters decayed notably (Satta et al., 2000).

**Table 2:** Isolates’ clade membership.

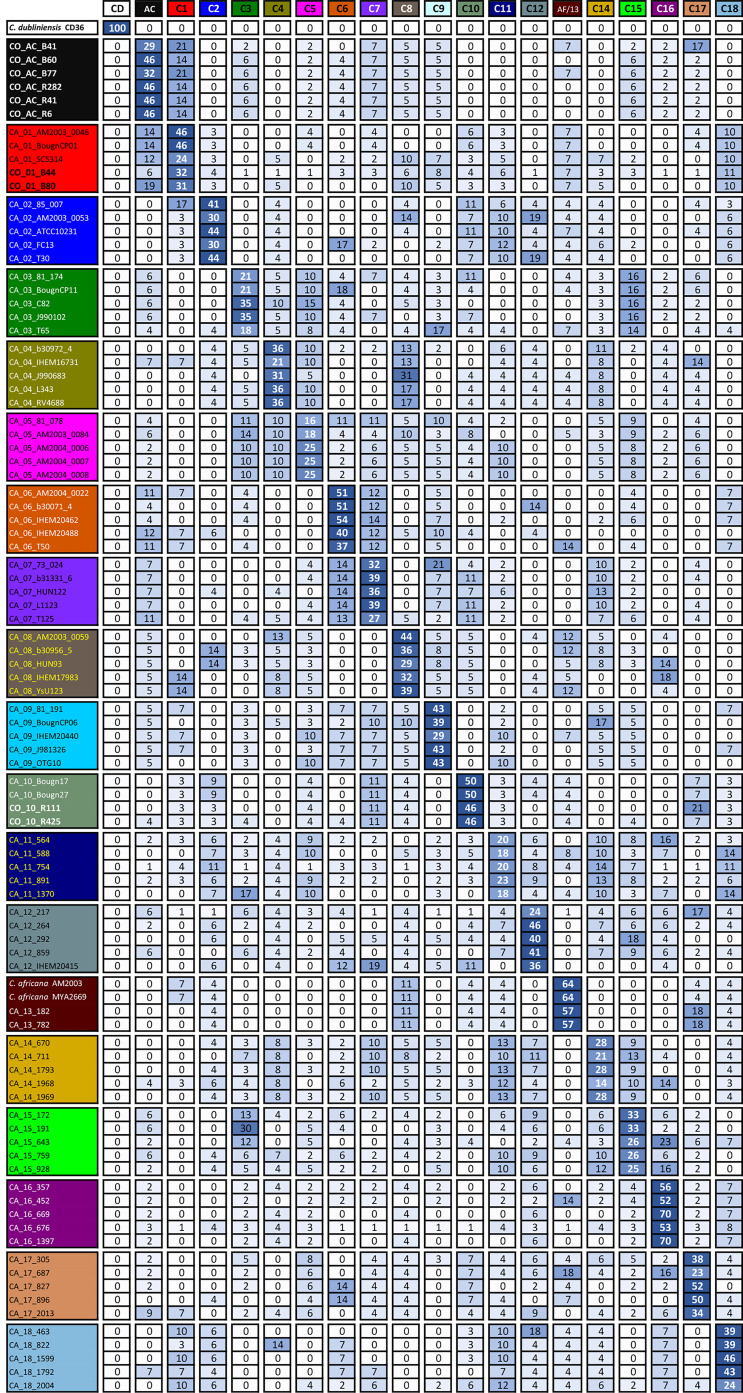	

An isolate’s consolidated membership is shown as a percentage. Large numbers denote predominant membership.

It has been observed that single-gene analysis has revealed incompatible association patterns (Rokas et al., 2003). However, it was observed here that clusters supported in MLST formed by sequences tending to be preferentially associated, regardless of the gene being considered and those having less than 95% support, contained isolates having associations with multiple clades (**Table 2**).

Consolidated isolate membership (**Table 2**) showed that they had a fuzzy assignment, having associations unnoticed in MLST cluster analysis. This fuzziness was observed for all clusters and separate membership analysis for each locus (**Supplementary Tables A to G**) showed that no clade consisted of perfectly differentiated isolates for all loci analysed in *C. albicans*, indicating that all groups could have shared alleles amongst different loci. Although classifications based on individual genes may have been incompatible with gene concatenation classification, combining the informational positions of all loci considered in the total analysis led to classification supported by well-differentiated clusters. AC isolates had strong stability in AC clusters, alternating their membership, mainly with clade 1 (and *vice versa*). The clustering pattern could be explained as a combined effect of random sorting of alleles, recombination events, and genetic drift (Satta et al., 2000; Rokas et al., 2003).

Atypical isolates in clade 1 had high membership in this clade, as did atypical isolates in clade 10 (**Table 2**). The SYA1 marker showed atypical isolates B41, B77 (AC cluster) and B44 and B80 (clade 1) association with *C. africana* (**Supplementary Figure** and **Table E**). The ACC1 marker had an association with atypical isolates R111 and R425 (clade 10) and *C. africana* (**Supplementary Figure** and **Table B**).

### Identifying Clone Complexes

**Figure 3:**
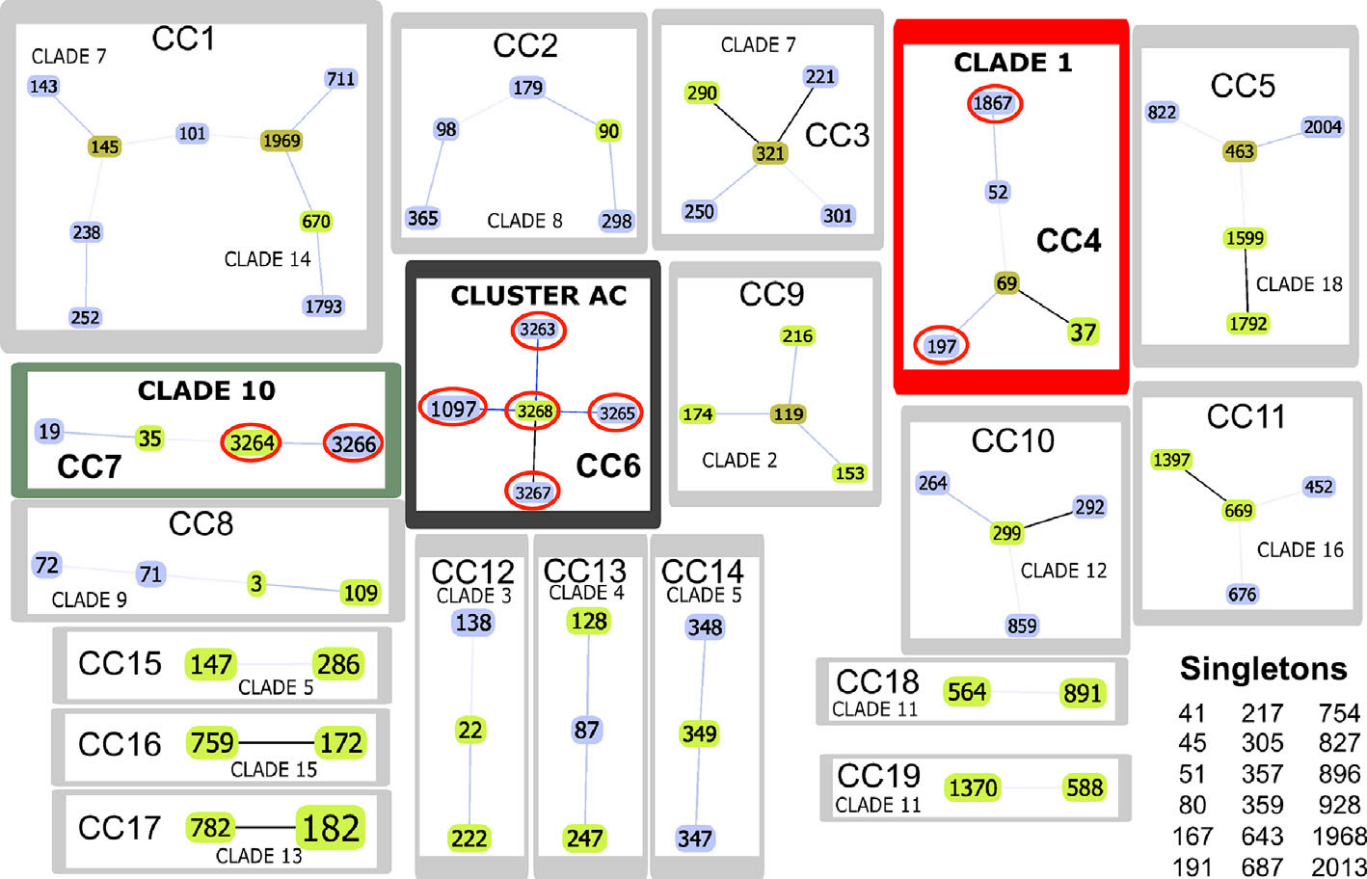
goeBURST analysis. The Colombian atypical isolates (red line ovals) were found in CC4 (framed in red), CC6 (black frame) and CC7 (green frame). Link colours: black links drawn without recourse to tiebreak rules; blue links drawn using tiebreak rule 1 (amount of single locus variations (SLV)); grey links drawn regarding triple locus variation (TLV) (lighter grey). Sequence type (ST) node colours: light green - founder group; dark green - founder sub-group; light blue - common node.

Nineteen clone complexes (CC) and 18 singletons were identified after using the goeBURST algorithm regarding allele profiles for the only 91 DSTs in the data set analysed here. CC1 included more DSTs formed by members of clades 7 and 14. It was found that most clades formed independent CC, confirming this strategy’s usefulness for describing the relationships between related DSTs. It was ascertained that atypical Colombian isolates were located in CC 4 (B44, B80), 7 (R11, R425), and 6 (R6, R282, B60, B77, R41, B41), the first two from clades 1 and 10 and the third from the isolates in the AC (**Figure 3** and **Table 1**).

### Killing Assays in *G. mellonella*

**Figure 4:**
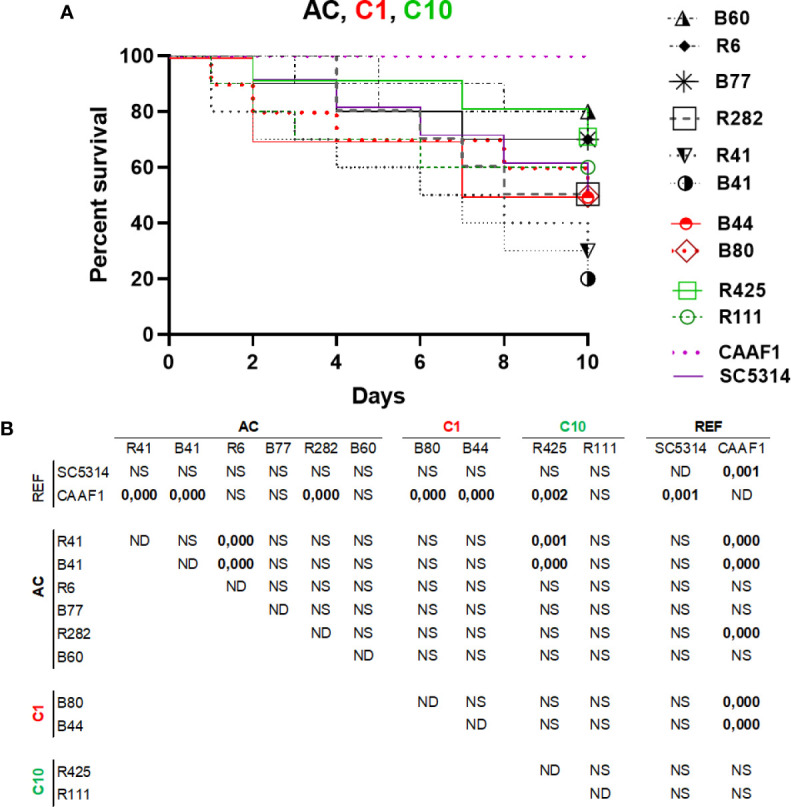
Pathogenicity in a *G*. *mellonella* infection model. **(A)** A Kaplan–Meier plot of *G. mellonella* survival after injection with 10^5^ CFU/larvae at 37°C. The data is expressed as survival percentage. No larval killing was observed in control larvae injected with an equivalent volume of PBS (data not shown). **(B)** log-rank (Mantel-Cox) statistical comparison of each survival curve. Data is representative of three independent experiments. Atypical clade shown in black, clade 1 red and clade 10 green.

*C. albicans* (SC5314)*, C. africana* (CAAF1), and atypical isolates’ pathogenicity was compared in a systemic infection model (i.e. the *G. mellonella* insect larvae model). The 10^5^ CFU per larva concentration had significant differences in some strains evaluated. *C. albicans* R41 and B41 were the most virulent strains, both being members of the AC; conversely, larval killing by *C. africana* isolates was significantly slower than that observed with *C. albicans* in larvae, having statistically significant results (**Figure 4A**). All the strains had similar pathogenicity to the *C. albicans* SC5314 refence strain, lacking statistical significance (*p* > 0.05). Nevertheless, the R41, R11, R282, R435, B41, B44, and B80 strains had statistical differences regarding pathogenicity profiles compared to the *C. africana* CAAF1 reference strain; regarding pathogenicity, it was evident that *C. africana* could not kill the larvae on the days evaluated here, demonstrating this species’ inability compared to that of *C. albicans* (**Figure 4B**). Concerning pathogenicity by clade, AC clade R41 and B41 strains were the only strains having statistically significant differences regarding the other *C. albicans* strains. The differences concerned members of the same clade (AC) and one member of the C10 Clade. Concerning the AC clade, 3/6 strains had differences from CAAF1. As for clade C1, both members (2/2) only had statistical differences regarding CAAF1. The C10 clade had 1/2 members having differences regarding the CAAF1, R41, and B41 strains (**Figure 4B**).

### Antifungal Susceptibility

The susceptibility results showed that all the reference strains were susceptible to the antifungal drugs tested here, highlighting the susceptibility tests’ inability to differentiate between species (*C. albicans, C. africana*) in ATCC yeasts. The results for the ten atypical strains were grouped into three clades (AC, 1 and 10). Reference strains’ susceptibility results were homogeneous, having no evidence of species differentiation or clade association. All of them were susceptible to CAS and AMB; none had resistance or dose-dependent susceptibility. Atypical *Candida* had a slightly increased FLU MIC and the B44 strain (clade 1) was resistant (**Table 3**).

**Table 3:** Atypical *C. albicans* antifungal activity.

	ID	Clade	Minimum inhibitory concentration (μg/ml)		
			Fluconazole	Amphotericin	Caspofungin
			24 h	24 h	24 h
Reference strains	*C. africana* (CAAF1)	13	0.19	0.025	0.016
	*C. africana (*MYA2669)	13	0.025	<0.002	0.002
	*C. albicans* (ATCC90028)	N/A	0.5	<0.002	0.032
	*C. albicans* (SC5314)	1	1.5	0.75	0.016
Atypical isolates	R6	AC	1	<0.002	<0.002
	B41	AC	1	0.016	<0.002
	B77	AC	2	0.032	0.08
	R282	AC	1	0.064	0.094
	R41	AC	0.038	0.016	<0.002
	B60	AC	2	<0.02	0.19
	B80	1	1.5	0.032	<0.002
	B44	1	>256*	0.004	0.032
	R425	10	2	0.032	0.12
	R111	10	1.5	0.016	<0.002

*Resistance.

## Discussion

Finding genetic markers for devising an approach involving patterns enabling transcending *C. albicans* epidemiology and predicting treatment protocols for this mycosis is one of the challenges in understanding its dynamics as a pathogen (Alanio et al., 2017). This work thus involved the molecular study of ten isolates having atypical phenotypical characteristics (Rodríguez-Leguizamón et al., 2015b). The study’s main objective was to determine how they were grouped regarding other isolates reported in the MLST database; an algorithm was thus designed for evaluating the robustness of the clades described to date, finding the groupings described in clades 1 and 10 as well as a well-differentiated cluster which was named AC.

Classification analysis of atypical isolates concerning the isolates characteristic of the 18 clades described for *C. albicans* showed that most atypical isolates (six of them: **B41, B60, B77, R282, R41, and R6**) concurred in a well-supported cluster (AC) (**Figure 2**) and also constituted CC6 (**Table 1**, **Figure 3**). These isolates had common ST for markers ACC1, ADP1, MPI, VPS13, and ZWF1b markers (**Table 1**), concurring in cluster analysis as a whole and regarding individual genes (**Table 2**, **Supplementary Figure**/**Tables A to G**). Isolate source did not seem to be a defining item, even though three of the isolates had urinary tract as source and two bronco-alveolar lavage (**Table 1**). DST 1097 (characterised by the five ST shared for this group) was observed in KW2558/11 (Kuwait, 2011. Source: blood), LH1-225 (USA, 2008), and XA14 isolates (China, 2007. S: oral swab) reported in the PubMLST database (Jolley et al., 2018). DST 590 was observed in KW106/13 (Kuwait, 2013, S: blood), KW150/13 (Kuwait, 2013. S: blood) and CL4752 isolates (Venezuela. S: blood), sharing four ST with the AC (ACC1, ADP1, VPS13 and ZWF1b) and the AAT1a marker, which had ST 13 that was common for 5/6 isolates in this group. The exception was B41 (**Table 1**), being the closest geographical reference for these Colombian isolates. ST diversity for the SYA1 marker in the atypical Colombian isolates was greater than that observed for its equivalent in isolates reported to date (**Table 1**), meaning that 4/6 AC isolates had unique DST.

Isolates B44 and B80 were associated with clade 1, having significant support and high co-occurrence (**Figure 2**, **Table 2**). The AC and clade 1 were related and their isolates were co-grouped in cluster analysis for individual markers (**Supplementary Figures A to G**). Isolate source was not a common defining characteristic and only 2 markers shared common ST (SYA1 and ZWF1b). The isolates analysed from clade 1 formed CC4 (**Table 1**, **Figure 3**). Isolate B44 had DST 1867 which was similar to thirty-five isolates previously reported in PubMLST (https://pubmlst.org/); all of them had exclusive geographical origin in China and vaginal swab as source, the same source as isolate B44. Isolate B80 had DST 197, similar to isolates AM2003/0073 (UK, 2003. S: blood) and BK04417 (Germany, 2008. S: blood).

The remaining atypical isolates, R111 and R425, were associated with isolates from clade 10 in a supported cluster, showing that all the members had high co-occurrence (**Figure 2**, **Table 2**, **Supplementary Figures** and **Tables A–G**) and formed CC7 (**Figure 3**). The group was defined by having three ST markers in common (ACC1, SYA1, VPS13) and both atypical samples had been isolated from urinary tracts (**Table 1**). The two atypical isolates in clade 10 differed regarding *AAT1a* and *MPIb* ST. R111 had the only DST 3264, sharing six markers with DST 1363 (except for ZWF1b from ST 14), comprising six isolates already reported in PubMLST: M15 and M16 (Morocco, 2008. S: vaginal swab), KW575/12, KW78/12, and KW98/12 (Kuwait, 2012. S: blood), and TW-CDC514 (Taiwan, 2000). R425 had the only DST 3266, differing regarding markers with DST 1362 (AAT1a = ST4, ZWF1b = ST14), being similar to isolate M14 (Morocco, 2008. S: vaginal swab).

Great similarity between both approaches was found when comparing e-BURST and cluster analysis. The amount of clonal complexes (CC) was very close to the amount of clusters (19 and 18, respectively), differences concerning the fusion of clades 7 and clade 14 in CC1, the separation of clades 5 and 11 (into CC15 - CC14 and CC18 and CC19, respectively) and 18 strains assigned as singletons (**Table 1** and **Figure 3**). Both analyses revealed the same association pattern for atypical isolates, identifying three well-defined classes.

Our group has described that atypical isolates share phenotypical features with *C. africana* even though analysis with molecular markers has identified these isolates as *C. albicans* (Rodríguez-Leguizamón et al., 2015b). Our results from analysing MLST data have shown that these isolates could be classified as *C. albicans*. A relationship between the atypical isolates and *C. africana* was only found concerning isolates B41 and B77 from the AC and isolates B44 and B80 from clade 1 with the SYA1 marker and isolates R111 and R425 from clade 10 with the ACC1 marker; this was not sufficient for classifying these atypical isolates as *C. africana*.

Atypical isolates’ pathogenic capability regarding *C. africana* in the *G. mellonella* model was different. *C. africana* did not cause larval mortality; interestingly, isolates R41 and B41 belonging to the AC and CC6 had greater virulence, demonstrating variability regarding their performance (**Figure 4**). It has been reported that *C. africana* cannot survive in the haemolymph along with high *G. mellonella* antimicrobial peptide (AMP) concentrations. Virulence factors have been extensively studied in *C. albicans* and include adhesins (ALS), enzymes (e.g. SAPS, PLA, PLB, PLC) and, notably, the ability to alternate between hyphal and budding yeast forms. No proteomics approach to date has deciphered the particularities concerning *C. albicans* and *C. africana*. It has been proposed that hyphal formation plays a crucial role in binding to host cell surface, tissue invasion, biofilm formation, and immune evasion. Alterations associated with delayed hyphal formation (possibly associated with different *HWP1* gene size), the absence of chlamydospores and the loss of enzyme battery could have reduced an ability to tolerate environmental stress, resulting in reduced *Galleria* larval virulence, as reduced *C. dubliniensis* virulence compared to that for *C. albicans* has been attributed to lower filamentation rates (Borman et al., 2013). *C. africana* was avirulent in the present study, as has been described in other studies regarding this species. It was observed that many isolates had a pathogenicity profile similar to that observed in the C. albicans SC5314 reference strain, despite having an atypical phenotype.

Atypical isolates conserved their pathogenic capability and had different fluconazole MIC values and resistance cut-off points were identified regarding the B44 strain (**Table 3**). Such results highlighted these yeasts’ great plasticity and ratified the difficulty involved in determining patterns in *Candida*. The study’s results enabled completing details regarding these isolates’ preliminary characterisation, selective pressure complexity, host supply and microenvironment diversity in hospital conditions. They also highlighted the need for the detailed phenotypical and functional characterisation of the atypical isolates studied here.

## Conclusion

The proposed classification analysis for the atypical isolates characterised here identified isolates belonging to *C. albicans*, in spite of having phenotypical characteristics coinciding with those for *C. africana*. Most were new DSTs (6/10); all were related to DST reported for regions geographically remote from Colombia, underlining circulating strains’ global dispersion or, less probably, MLST variants convergence between isolates from Colombia, northern Africa, North America, Europe and Asia. The AC’s distinctive characters suggested its relevance as a new clade for *C. albicans*. The AC has been consistently differentiated from the clades reported to date by MLST characterisation, trehalose metabolism and an inability to form chlamydospores, unlike typical *C. albicans* (Rodríguez-Leguizamón et al., 2015b).

This study concluded that most atypical isolates belonged to *C. albicans* species and represented new DSTs, or came within DSTs reported in distant geographical regions. The isolates were clearly differentiated from *C. africana* regarding individual markers and concatenation, except for isolates having ST 2 in the *SYA1* gene (B41, B77, B44 and B80) and ST 7 in the *ACC1* gene (R111 and R425) shared by isolates from clade 13 to which *C. africana* belongs (**Table 1**, **Supplementary Figures A to G**).

The proposed classification methodology enabled hierarchical clustering using unbiased Bootstrap as statistical support for MLST characterisation together and separately, clearly recovering established clades (Bougnoux et al., 2004; McManus and Coleman, 2014). It offered an image coinciding with *C. albicans*’ complex population structure where classification patterns highlighted the dynamic nature of this pathobiont’s populations. The *C. albicans* population’s genetic structure is extremely heterogeneous and great variation could be observed here regarding the descriptions available in the pertinent medical literature, consistently grouping clinical isolates sharing complex hospital environments.

The phenotypical patterns described in our group’s studies have been close to the descriptions in the literature about phenotypical plasticity really affecting features such as the virulence seen in both strains from the AC in the *G. mellonella* model and another strain from clade 1 that had fluconazole resistance. This type of finding highlights the need for more studies in this field as a response to the challenge of difficult-to-diagnose and manage hospital-acquired infections.

## Data Availability Statement

The raw data supporting the conclusions of this article will be made available by the authors, without undue reservation.

## Ethics Statement

The studies involving human participants were reviewed and approved by the Universidad del Rosario’s ethics committee and the Hospital San Ignacio’s ethics committee.

## Author Contributions

GR-L: conceived the study, participated in its design, participated in acquiring MLST data and analysed it. AC-G: performed killing assays in *G. mellonella* and susceptibility tests. CS: designed, carried out and discussed the cluster and membership analysis of MLST. MP: coordinated acquiring MLST data, participated in its design and critically reviewed the manuscript. CP-G: coordinated the study and participated in its design. All authors contributed to the article and approved the submitted version.

## Funding

This study was funded by the Fundación Instituto de Inmunología de Colombia (FIDIC), the Universidad del Rosario and Universidad Javeriana, Ponticia Universidad Javeriana ID006878 Call 777/2017 MinCiencias. Hospital Universitario Mayor ID 24668.

## Conflict of Interest

The authors declare that the research was conducted in the absence of any commercial or financial relationships that could be construed as a potential conflict of interest.

## Supplementary Material

Supplementary Figure 1 **(A–G)** Cluster analysis of separated MLST markers using the UPGMA method. Each clade is shown in a different colour. Colombian isolates are indicated in bold and chevrons. Bootstrap values above 95% were considered significant. Large numbers in red denote support for each clade. TIP, total informative positions; CAIP, *C. albicans* informative positions.

Supplementary Table 1 **(A–G)** Isolates’ clade membership for each MLST marker. An isolate’s membership is shown as a percentage. Values close to 1 denote predominant membership.

## Abbreviations

AC, atypical cluster; AMB, amphotericin B; CAIP, *C. albicans* informative position; CAS, caspofungin; CC, clone complex; DST, diploid sequence type; FLU, fluconazole; IFD, invasive fungal disease; MALDI-TOF-MS, matrix-assisted laser desorption/ionization time-of-flight mass spectrometry; MIC, minimal inhibitory concentration; MLST, multilocus sequence typing; PBS, phosphate-buffered saline; RPMI, Roswell Park Memorial Institute; TIP, total informative position; UPGMA, unweighted pair group method with arithmetic mean.
