# Supplementary material for: Robust, Comprehensive Molecular, and Phenotypical Characterisation of Atypical Candida albicans Clinical Isolates From Bogotá, Colombia
Source: Front Cell Infect Microbiol. 2020 Dec 2;10:571147. doi: 10.3389/fcimb.2020.571147 (PMC7738613; doi:10.3389/fcimb.2020.571147)
Supplement: Supplementary Figure 1 — (A–G) Cluster analysis of separated MLST markers using the UPGMA method. Each clade is shown in a different colour. Colombian isolates are indicated in bold and chevrons. Bootstrap values above 95% were considered significant. Large numbers in red denote support for each clade. TIP, total informative positions; CAIP, C. albicans informative positions. [file DataSheet_1.pdf]

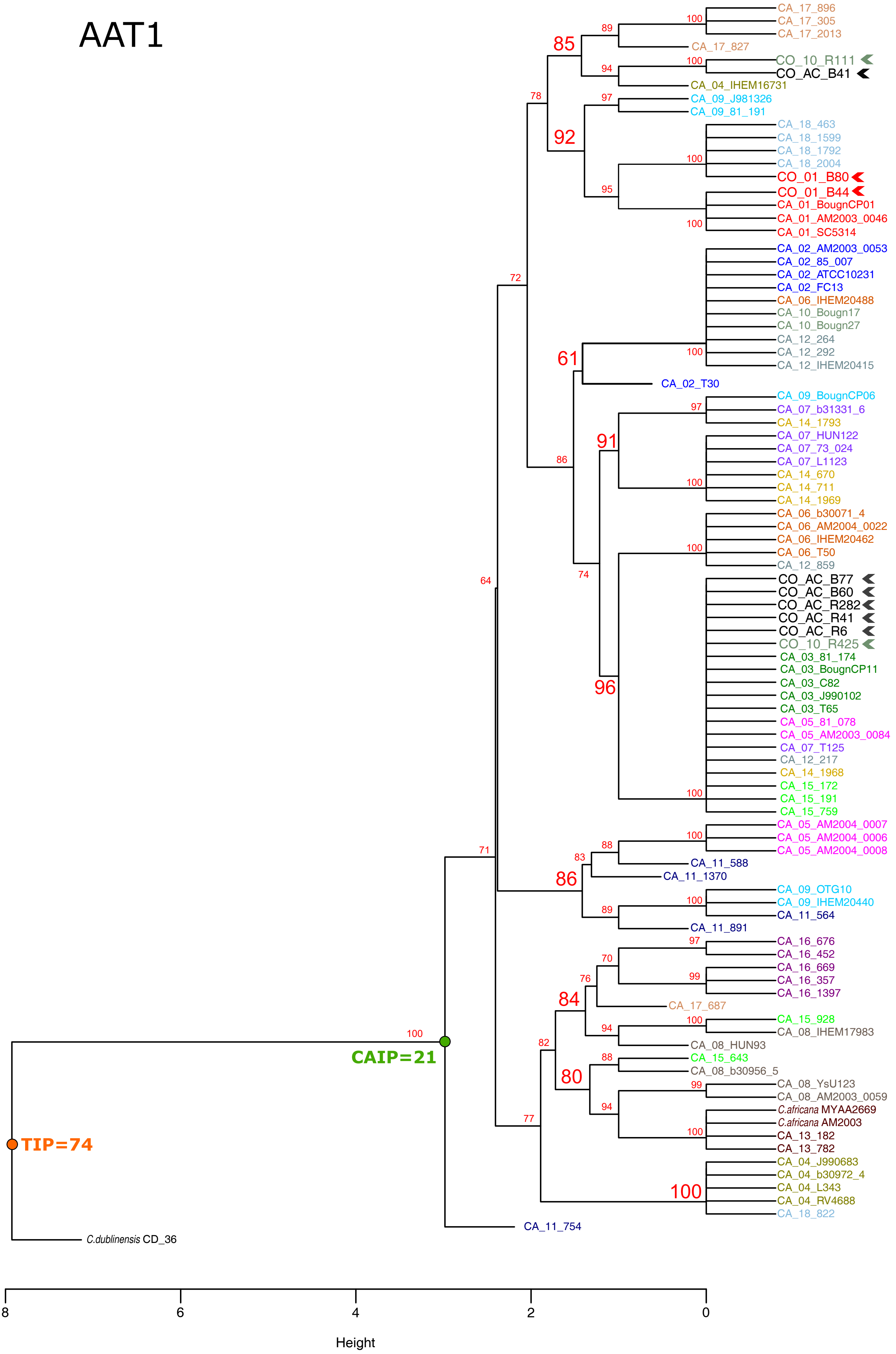

Supplementary Figure A

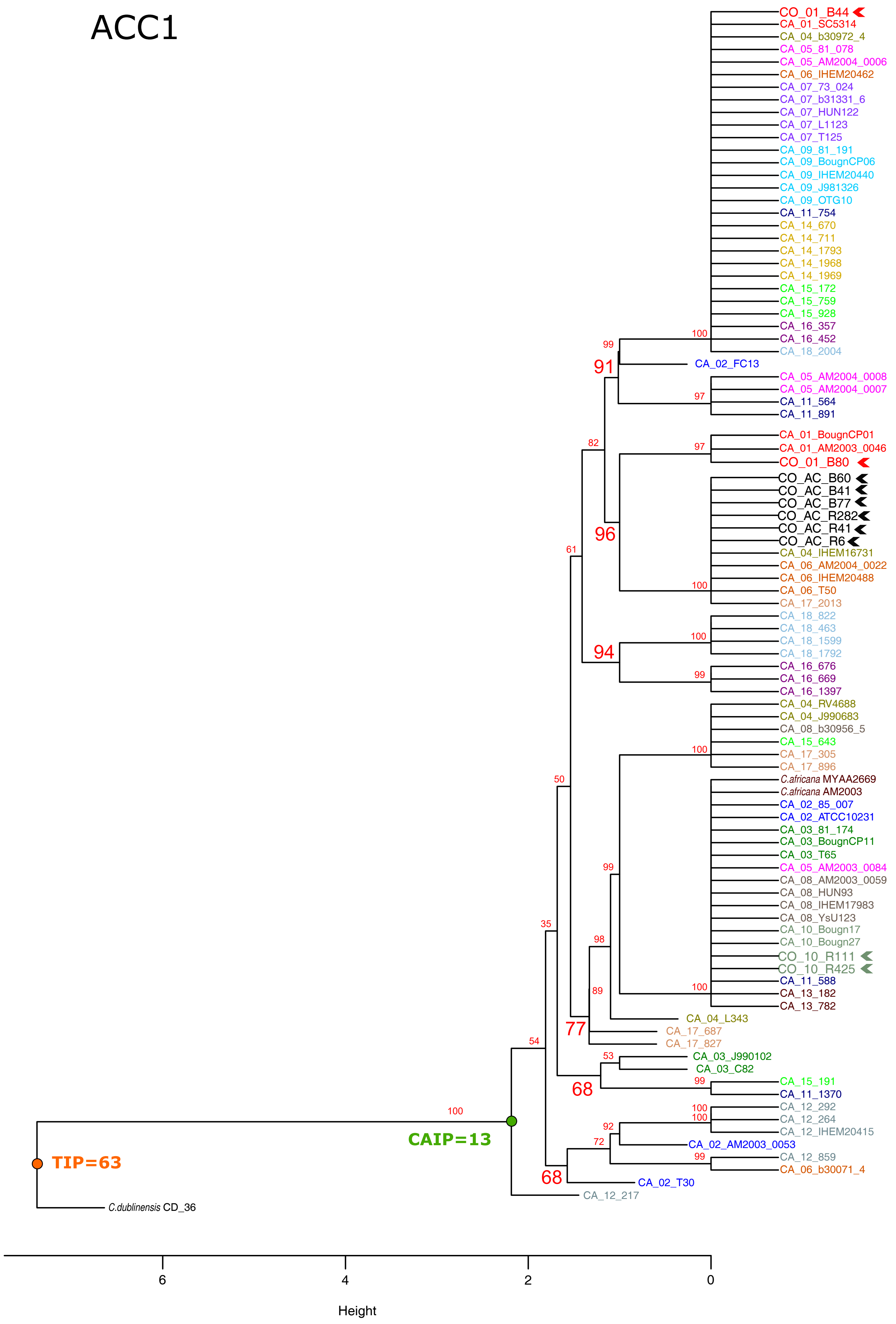

Supplementary Figure B

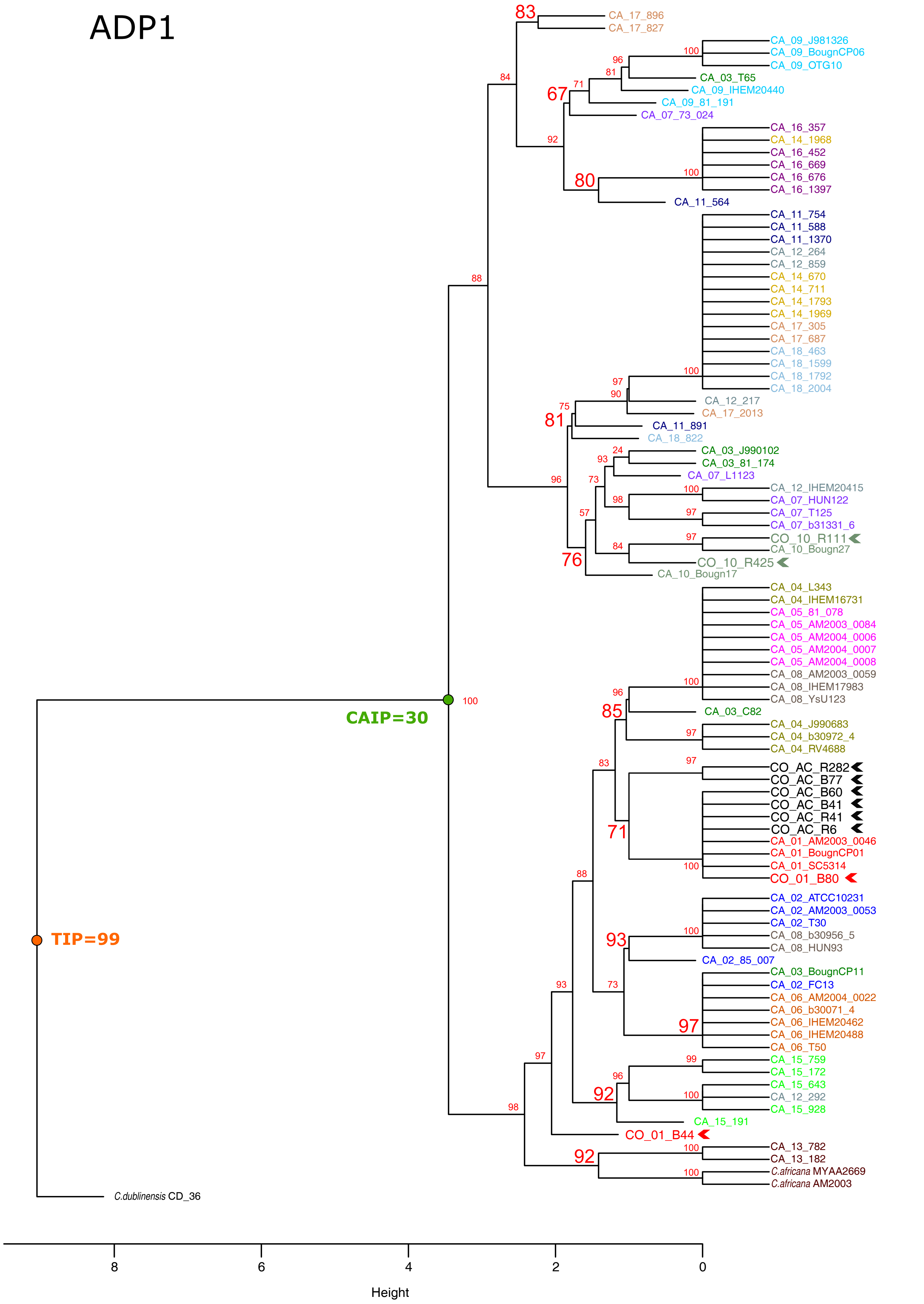

Supplementary Figure C

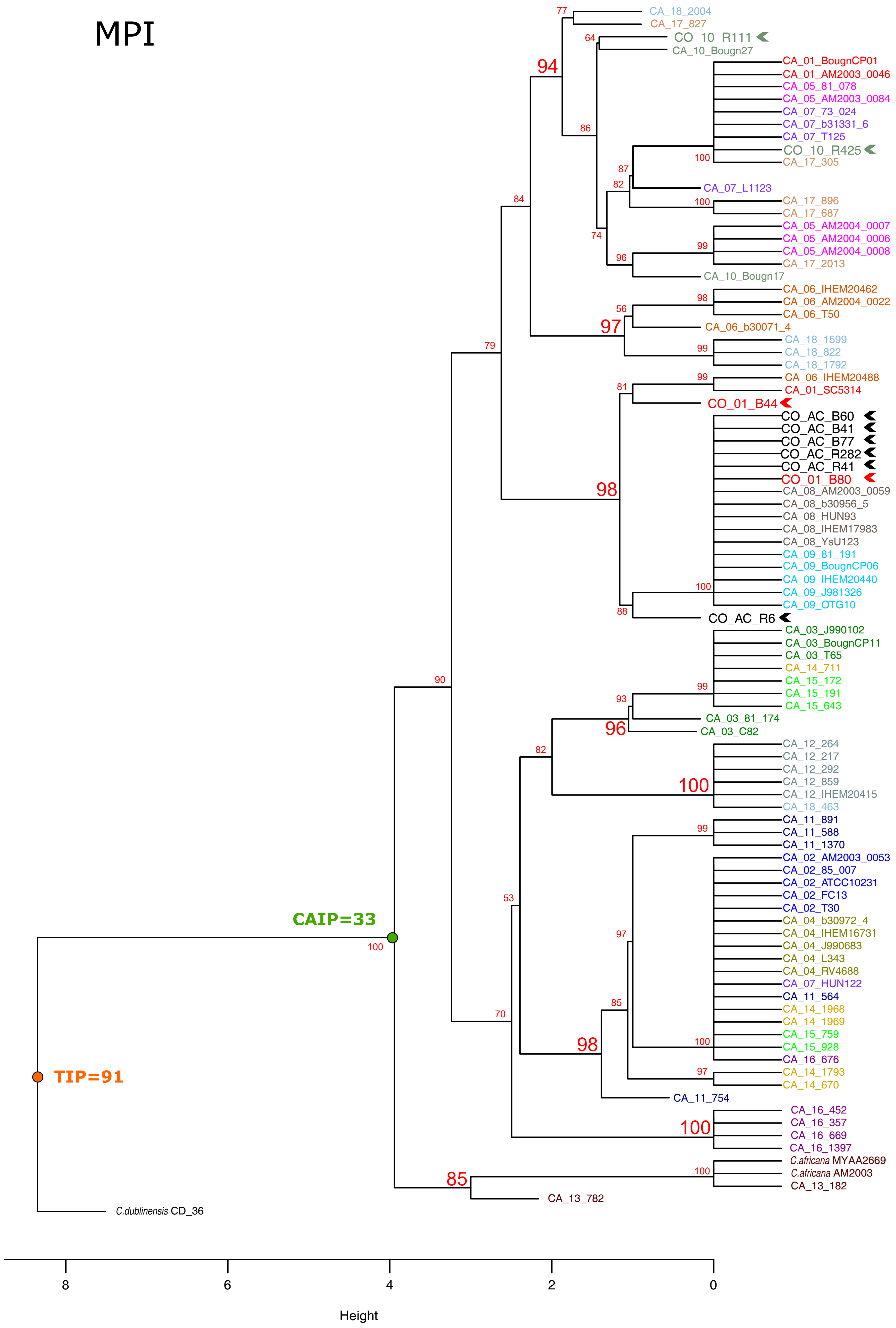

Supplementary Figure D

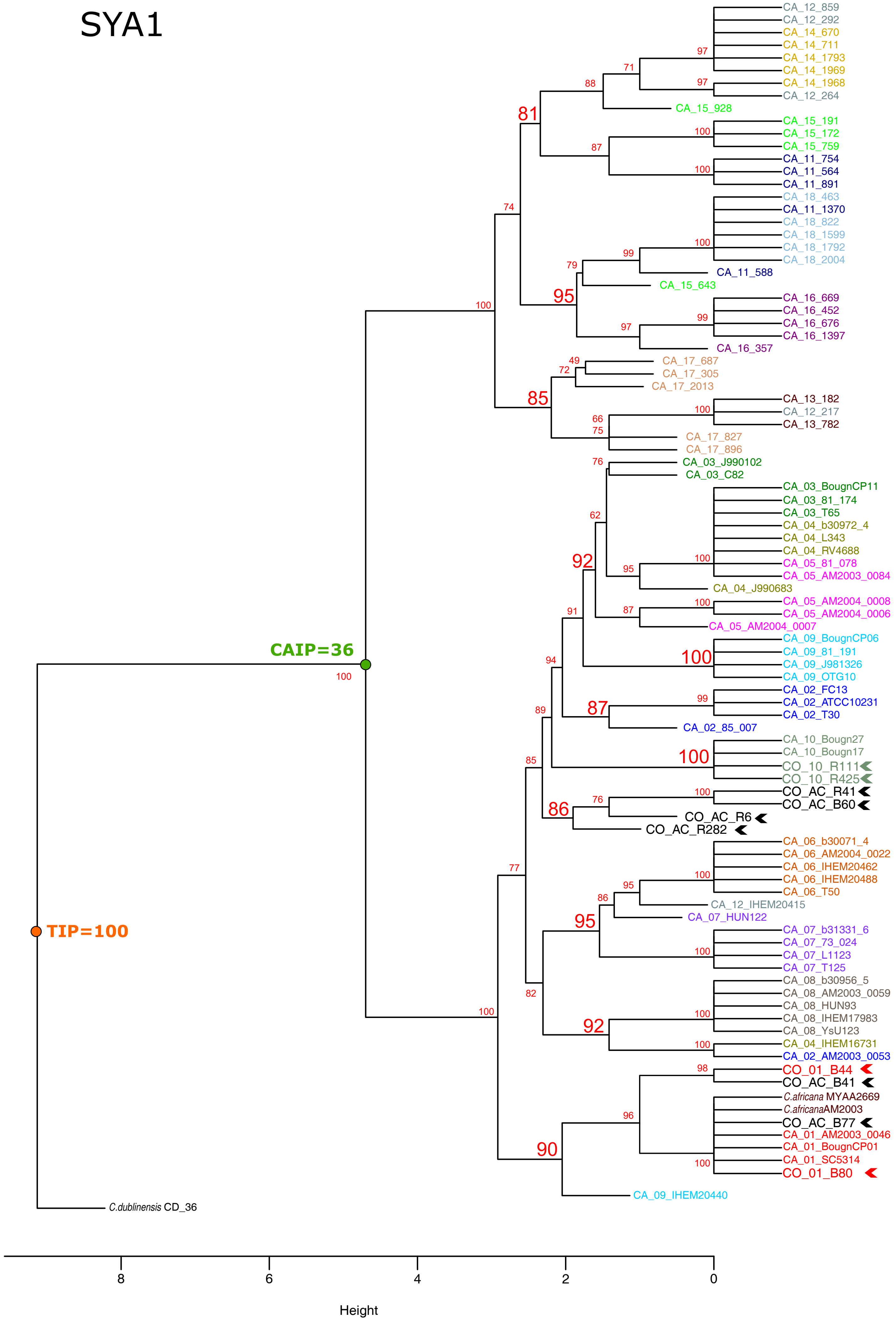

Supplementary Figure E

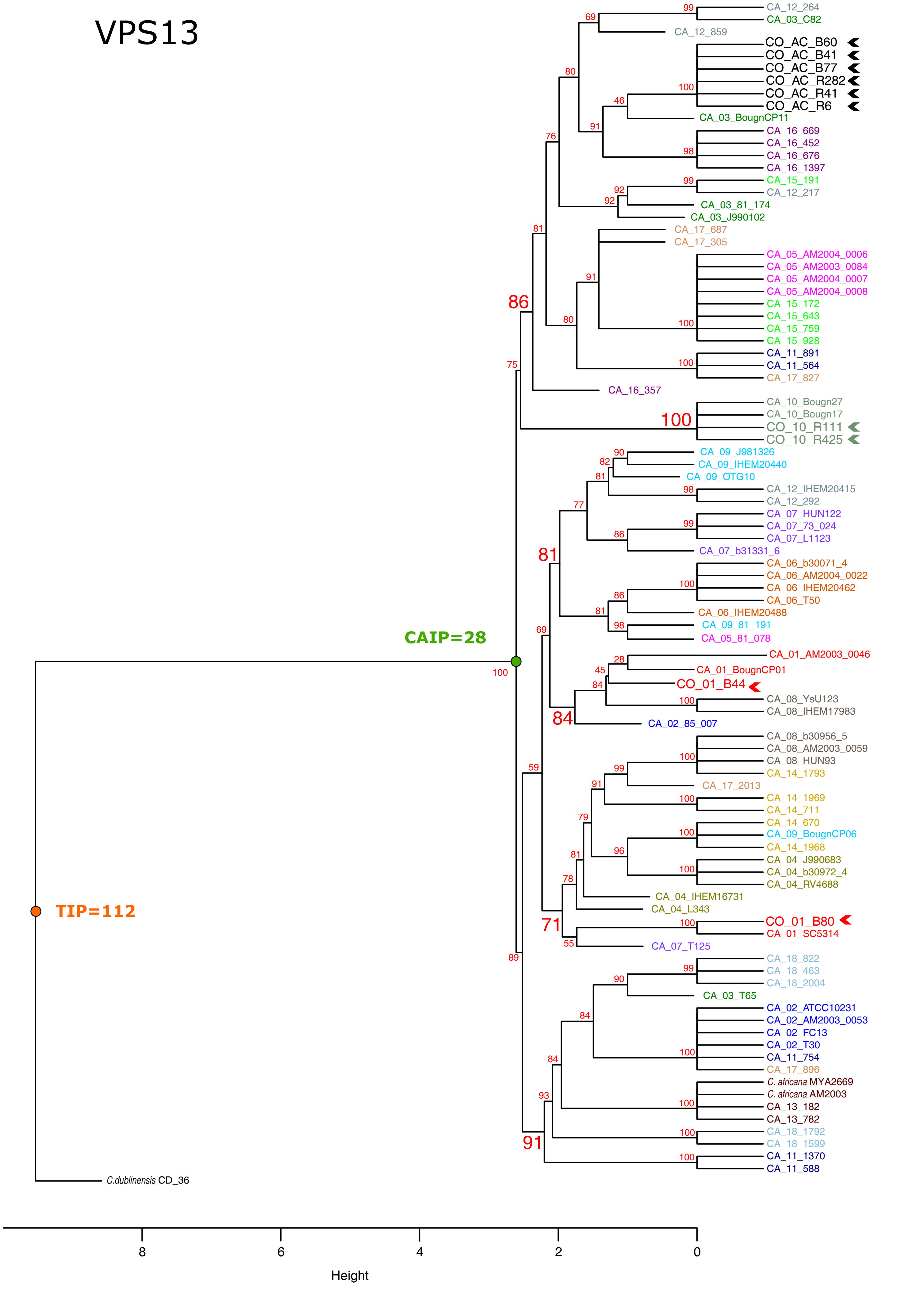

Supplementary Figure F

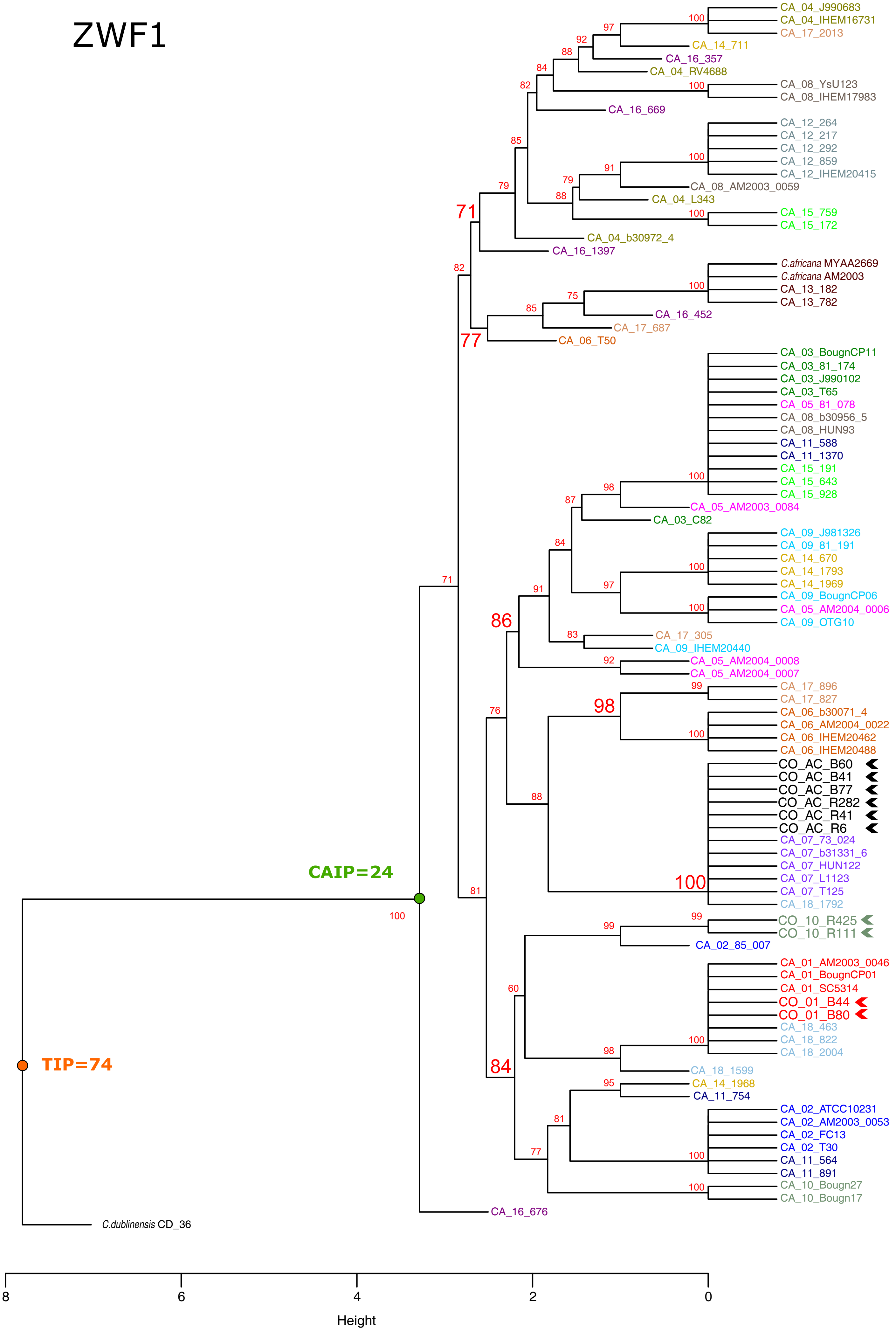

Supplementary Figure G

Supplementary Table A

Isolate's membership to *C. albicans* clades based on gene AAT1

	CD	AC	C1	C2	C3	C4	C5	C6	C7	C8	C9	C10	C11	C12	AF/13	C14	C15	C16	C17	C18
C. dubliniensis CD36	1.00																			
**CO_AC_B41**																			1.00	
**CO_AC_B60**		0.25			0.25			0.25									0.25			
**CO_AC_B77**		0.25			0.25			0.25									0.25			
**CO_AC_R282**		0.25			0.25			0.25									0.25			
**CO_AC_R41**		0.25			0.25			0.25									0.25			
**CO_AC_R6**		0.25			0.25			0.25									0.25			
CA_01_AM2003_0046			0.50																	0.50
CA_01_BougnCP01			0.50																	0.50
CA_01_SC5314			0.50																	0.50
**CO_01_B44**			0.50																	0.50
**CO_01_B80**			0.50																	0.50
CA_02_85_007				0.40								0.30		0.30						
CA_02_AM2003_0053				0.40								0.30		0.30						
CA_02_ATCC10231				0.40								0.30		0.30						
CA_02_FC13				0.40								0.30		0.30						
CA_02_T30				0.40								0.30		0.30						
CA_03_81_174		0.25			0.25			0.25									0.25			
CA_03_BougnCP11		0.25			0.25			0.25									0.25			
CA_03_C82		0.25			0.25			0.25									0.25			
CA_03_J990102		0.25			0.25			0.25									0.25			
CA_03_T65		0.25			0.25			0.25									0.25			
CA_04_b30972_4						1.00														
CA_04_IHEM16731																			1.00	
CA_04_J990683						1.00														
CA_04_L343						1.00														
CA_04_RV4688						1.00														
CA_05_81_078		0.25			0.25			0.25									0.25			
CA_05_AM2003_0084		0.25			0.25			0.25									0.25			
CA_05_AM2004_0006							0.50						0.50							
CA_05_AM2004_0007							0.50						0.50							
CA_05_AM2004_0008							0.50						0.50							
CA_06_AM2004_0022		0.25			0.25			0.25									0.25			
CA_06_b30071_4		0.25			0.25			0.25									0.25			
CA_06_IHEM20462		0.25			0.25			0.25									0.25			
CA_06_IHEM20488				0.40								0.30		0.30						
CA_06_T50		0.25			0.25			0.25									0.25			
CA_07_73_024									0.50							0.50				
CA_07_b31331_6									0.50							0.50				
CA_07_HUN122									0.50							0.50				
CA_07_L1123									0.50							0.50				
CA_07_T125		0.25			0.25			0.25									0.25			
CA_08_AM2003_0059										0.50					0.50					
CA_08_b30956_5										0.50					0.50					
CA_08_HUN93																		1.00		
CA_08_IHEM17983																		1.00		
CA_08_YsU123										0.50					0.50					
CA_09_81_191			0.50																	0.50
CA_09_BougnCP06									0.50							0.50				
CA_09_IHEM20440							0.50						0.50							
CA_09_J981326			0.50																	0.50
CA_09_OTG10							0.50						0.50							
CA_10_Bougn17				0.40								0.30		0.30						
CA_10_Bougn27				0.40								0.30		0.30						
**CO_10_R111**																			1.00	
**CO_10_R425**		0.25			0.25			0.25									0.25			
CA_11_564							0.50						0.50							
CA_11_588							0.50						0.50							
CA_11_754		0.05	0.05	0.05	0.05	0.05	0.05	0.05	0.05	0.05	0.05	0.05	0.05	0.05	0.05	0.05	0.05	0.05	0.05	0.05
CA_11_891							0.50						0.50							
CA_11_1370							0.50						0.50							
CA_12_217		0.25			0.25			0.25									0.25			
CA_12_264				0.40								0.30		0.30						
CA_12_292				0.40								0.30		0.30						
CA_12_859		0.25			0.25			0.25									0.25			
CA_12_IHEM20415				0.40								0.30		0.30						
C. africana AM2003										0.50					0.50					
C. africana MYA2669										0.50					0.50					
CA_13_182										0.50					0.50					
CA_13_782										0.50					0.50					
CA_14_670									0.50							0.50				
CA_14_711									0.50							0.50				
CA_14_1793									0.50							0.50				
CA_14_1968		0.25			0.25			0.25									0.25			
CA_14_1969									0.50							0.50				
CA_15_172		0.25			0.25			0.25									0.25			
CA_15_191		0.25			0.25			0.25									0.25			
CA_15_643																		1.00		
CA_15_759		0.25			0.25			0.25									0.25			
CA_15_928																		1.00		
CA_16_357																		1.00		
CA_16_452																		1.00		
CA_16_669																		1.00		
CA_16_676																		1.00		
CA_16_1397																		1.00		
CA_17_305																			1.00	
CA_17_687																		1.00		
CA_17_827																			1.00	
CA_17_896																			1.00	
CA_17_2013																			1.00	
CA_18_463			0.50																	0.50
CA_18_822						1.00														
CA_18_1599			0.50																	0.50
CA_18_1792			0.50																	0.50
CA_18_2004			0.50																	0.50

Supplementary Table B

Isolate's membership to *C. albicans* clades based on gene ACC1

	CD	AC	C1	C2	C3	C4	C5	C6	C7	C8	C9	C10	C11	C12	AF/13	C14	C15	C16	C17	C18
***C. dubliniensis* CD36**	1.00																			
**CO_AC_B41**		0.50	0.50																	
**CO_AC_B60**		0.50	0.50																	
**CO_AC_B77**		0.50	0.50																	
**CO_AC_R282**		0.50	0.50																	
**CO_AC_R41**		0.50	0.50																	
**CO_AC_R6**		0.50	0.50																	
CA_01_AM2003_0046		0.50	0.50																	
CA_01_BougnCP01		0.50	0.50																	
CA_01_SC5314								0.17	0.17		0.17		0.17			0.17	0.17			
**CO_01_B44**								0.17	0.17		0.17		0.17			0.17	0.17			
**CO_01_B80**		0.50	0.50																	
CA_02_85_007										0.25		0.25			0.25				0.25	
CA_02_AM2003_0053														1.00						
CA_02_ATCC10231										0.25		0.25			0.25				0.25	
CA_02_FC13								0.17	0.17		0.17		0.17			0.17	0.17			
CA_02_T30														1.00						
CA_03_81_174										0.25		0.25			0.25				0.25	
CA_03_BougnCP11										0.25		0.25			0.25				0.25	
CA_03_C82					1.00															
CA_03_J990102					1.00															
CA_03_T65										0.25		0.25			0.25				0.25	
CA_04_b30972_4								0.17	0.17		0.17		0.17			0.17	0.17			
CA_04_IHEM16731		0.50	0.50																	
CA_04_J990683										0.25		0.25			0.25				0.25	
CA_04_L343										0.25		0.25			0.25				0.25	
CA_04_RV4688										0.25		0.25			0.25				0.25	
CA_05_81_078								0.17	0.17		0.17		0.17			0.17	0.17			
CA_05_AM2003_0084										0.33		0.33			0.33					
CA_05_AM2004_0006								0.17	0.17		0.17		0.17			0.17	0.17			
CA_05_AM2004_0007								0.17	0.17		0.17		0.17			0.17	0.17			
CA_05_AM2004_0008								0.17	0.17		0.17		0.17			0.17	0.17			
CA_06_AM2004_0022		0.50	0.50																	
CA_06_b30071_4														1.00						
CA_06_IHEM20462								0.17	0.17		0.17		0.17			0.17	0.17			
CA_06_IHEM20488		0.50	0.50																	
CA_06_T50		0.50	0.50																	
CA_07_73_024								0.17	0.17		0.17		0.17			0.17	0.17			
CA_07_b31331_6								0.17	0.17		0.17		0.17			0.17	0.17			
CA_07_HUN122								0.17	0.17		0.17		0.17			0.17	0.17			
CA_07_L1123								0.17	0.17		0.17		0.17			0.17	0.17			
CA_07_T125								0.17	0.17		0.17		0.17			0.17	0.17			
CA_08_AM2003_0059										0.33		0.33			0.33					
CA_08_b30956_5										0.33		0.33			0.33					
CA_08_HUN93										0.33		0.33			0.33					
CA_08_IHEM17983										0.33		0.33			0.33					
CA_08_YsU123										0.33		0.33			0.33					
CA_09_81_191								0.17	0.17		0.17		0.17			0.17	0.17			
CA_09_BougnCP06								0.17	0.17		0.17		0.17			0.17	0.17			
CA_09_IHEM20440								0.17	0.17		0.17		0.17			0.17	0.17			
CA_09_J981326								0.17	0.17		0.17		0.17			0.17	0.17			
CA_09_OTG10								0.17	0.17		0.17		0.17			0.17	0.17			
CA_10_Bougn17										0.25		0.25			0.25				0.25	
CA_10_Bougn27										0.25		0.25			0.25				0.25	
**CO_10_R111**										0.25		0.25			0.25				0.25	
**CO_10_R425**										0.25		0.25			0.25				0.25	
CA_11_564								0.17	0.17		0.17		0.17			0.17	0.17			
CA_11_588										0.33		0.33			0.33					
CA_11_754								0.17	0.17		0.17		0.17			0.17	0.17			
CA_11_891								0.17	0.17		0.17		0.17			0.17	0.17			
CA_11_1370					1.00															
CA_12_217		0.05	0.05	0.05	0.05	0.05	0.05	0.05	0.05	0.05	0.05	0.05	0.05	0.05	0.05	0.05	0.05	0.05	0.05	0.05
CA_12_264														1.00						
CA_12_292														1.00						
CA_12_859														1.00						
CA_12_IHEM20415														1.00						
C. africana AM2003										0.25		0.25			0.25				0.25	
C. africana MYA2669										0.25		0.25			0.25				0.25	
CA_13_182										0.25		0.25			0.25				0.25	
CA_13_782										0.25		0.25			0.25				0.25	
CA_14_670								0.17	0.17		0.17		0.17			0.17	0.17			
CA_14_711								0.17	0.17		0.17		0.17			0.17	0.17			
CA_14_1793								0.17	0.17		0.17		0.17			0.17	0.17			
CA_14_1968								0.17	0.17		0.17		0.17			0.17	0.17			
CA_14_1969								0.17	0.17		0.17		0.17			0.17	0.17			
CA_15_172								0.17	0.17		0.17		0.17			0.17	0.17			
CA_15_191					1.00															
CA_15_643										0.25		0.25			0.25				0.25	
CA_15_759								0.17	0.17		0.17		0.17			0.17	0.17			
CA_15_928								0.17	0.17		0.17		0.17			0.17	0.17			
CA_16_357								0.17	0.17		0.17		0.17			0.17	0.17			
CA_16_452								0.17	0.17		0.17		0.17			0.17	0.17			
CA_16_669																		1.00		
CA_16_676																		1.00		
CA_16_1397																		1.00		
CA_17_305										0.25		0.25			0.25				0.25	
CA_17_687										0.25		0.25			0.25				0.25	
CA_17_827										0.25		0.25			0.25				0.25	
CA_17_896										0.25		0.25			0.25				0.25	
CA_17_2013		0.50	0.50																	
CA_18_463																				1.00
CA_18_822																				1.00
CA_18_1599																				1.00
CA_18_1792																				1.00
CA_18_2004								0.17	0.17		0.17		0.17			0.17	0.17			

Supplementary Table C

Isolate's membership to *C. albicans* clades based on gene ADP1

	CD	AC	C1	C2	C3	C4	C5	C6	C7	C8	C9	C10	C11	C12	AF/13	C14	C15	C16	C17	C18
***C. dubliniensis* CD36**	1.00																			
**CO_AC_B41**		0.50	0.50																	
**CO_AC_B60**		0.50	0.50																	
**CO_AC_B77**		0.50	0.50																	
**CO_AC_R282**		0.50	0.50																	
**CO_AC_R41**		0.50	0.50																	
**CO_AC_R6**		0.50	0.50																	
CA_01_AM2003_0046		0.50	0.50																	
CA_01_BougnCP01		0.50	0.50																	
CA_01_SC5314		0.50	0.50																	
**CO_01_B44**		0.06	0.06	0.06	0.06	0.06	0.06	0.06	0.06	0.06	0.06	0.06	0.06	0.06		0.06	0.06	0.06	0.06	0.06
**CO_01_B80**		0.50	0.50																	
CA_02_85_007				1.00																
CA_02_AM2003_0053				1.00																
CA_02_ATCC10231				1.00																
CA_02_FC13								1.00												
CA_02_T30				1.00																
CA_03_81_174									0.50			0.50								
CA_03_BougnCP11								1.00												
CA_03_C82						0.33	0.33			0.33										
CA_03_J990102									0.50			0.50								
CA_03_T65											1.00									
CA_04_b30972_4						0.33	0.33			0.33										
CA_04_IHEM16731						0.33	0.33			0.33										
CA_04_J990683						0.33	0.33			0.33										
CA_04_L343						0.33	0.33			0.33										
CA_04_RV4688						0.33	0.33			0.33										
CA_05_81_078						0.33	0.33			0.33										
CA_05_AM2003_0084						0.33	0.33			0.33										
CA_05_AM2004_0006						0.33	0.33			0.33										
CA_05_AM2004_0007						0.33	0.33			0.33										
CA_05_AM2004_0008						0.33	0.33			0.33										
CA_06_AM2004_0022								1.00												
CA_06_b30071_4								1.00												
CA_06_IHEM20462								1.00												
CA_06_IHEM20488								1.00												
CA_06_T50								1.00												
CA_07_73_024											1.00									
CA_07_b31331_6									0.50			0.50								
CA_07_HUN122									0.50			0.50								
CA_07_L1123									0.50			0.50								
CA_07_T125									0.50			0.50								
CA_08_AM2003_0059						0.33	0.33			0.33										
CA_08_b30956_5				1.00																
CA_08_HUN93				1.00																
CA_08_IHEM17983						0.33	0.33			0.33										
CA_08_YsU123						0.33	0.33			0.33										
CA_09_81_191											1.00									
CA_09_BougnCP06											1.00									
CA_09_IHEM20440											1.00									
CA_09_J981326											1.00									
CA_09_OTG10											1.00									
CA_10_Bougn17									0.50			0.50								
CA_10_Bougn27									0.50			0.50								
**CO_10_R111**									0.50			0.50								
**CO_10_R425**									0.50			0.50								
CA_11_564																		1.00		
CA_11_588													0.25	0.25		0.25				0.25
CA_11_754													0.25	0.25		0.25				0.25
CA_11_891													0.25	0.25		0.25				0.25
CA_11_1370													0.25	0.25		0.25				0.25
CA_12_217													0.25	0.25		0.25				0.25
CA_12_264													0.25	0.25		0.25				0.25
CA_12_292																	1.00			
CA_12_859													0.25	0.25		0.25				0.25
CA_12_IHEM20415									0.50			0.50								
C. africana AM2003															1.00					
C. africana MYA2669															1.00					
CA_13_182															1.00					
CA_13_782															1.00					
CA_14_670													0.25	0.25		0.25				0.25
CA_14_711													0.25	0.25		0.25				0.25
CA_14_1793													0.25	0.25		0.25				0.25
CA_14_1968																		1.00		
CA_14_1969													0.25	0.25		0.25				0.25
CA_15_172																	1.00			
CA_15_191																	1.00			
CA_15_643																	1.00			
CA_15_759																	1.00			
CA_15_928																	1.00			
CA_16_357																		1.00		
CA_16_452																		1.00		
CA_16_669																		1.00		
CA_16_676																		1.00		
CA_16_1397																		1.00		
CA_17_305													0.25	0.25		0.25				0.25
CA_17_687													0.25	0.25		0.25				0.25
CA_17_827																			1.00	
CA_17_896																			1.00	
CA_17_2013													0.25	0.25		0.25				0.25
CA_18_463													0.25	0.25		0.25				0.25
CA_18_822													0.25	0.25		0.25				0.25
CA_18_1599													0.25	0.25		0.25				0.25
CA_18_1792													0.25	0.25		0.25				0.25
CA_18_2004													0.25	0.25		0.25				0.25

Supplementary Table D

Isolate's membership to *C. albicans* clades based on gene MPI

	CD	AC	C1	C2	C3	C4	C5	C6	C7	C8	C9	C10	C11	C12	AF/13	C14	C15	C16	C17	C18
C. dubliniensis CD36	1.00																			
CO_AC_B41		0.33								0.33	0.33									
CO_AC_B60		0.33								0.33	0.33									
CO_AC_B77		0.33								0.33	0.33									
CO_AC_R282		0.33								0.33	0.33									
CO_AC_R41		0.33								0.33	0.33									
CO_AC_R6		0.33								0.33	0.33									
CA_01_AM2003_0046							0.25		0.25			0.25							0.25	
CA_01_BougnCP01							0.25		0.25			0.25							0.25	
CA_01_SC5314		0.33								0.33	0.33									
CO_01_B44		0.33								0.33	0.33									
CO_01_B80		0.33								0.33	0.33									
CA_02_85_007				0.25		0.25							0.25			0.25				
CA_02_AM2003_0053				0.25		0.25							0.25			0.25				
CA_02_ATCC10231				0.25		0.25							0.25			0.25				
CA_02_FC13				0.25		0.25							0.25			0.25				
CA_02_T30				0.25		0.25							0.25			0.25				
CA_03_81_174					0.50												0.50			
CA_03_BougnCP11					0.50												0.50			
CA_03_C82					0.50												0.50			
CA_03_J990102					0.50												0.50			
CA_03_T65					0.50												0.50			
CA_04_b30972_4				0.25		0.25							0.25			0.25				
CA_04_IHEM16731				0.25		0.25							0.25			0.25				
CA_04_J990683				0.25		0.25							0.25			0.25				
CA_04_L343				0.25		0.25							0.25			0.25				
CA_04_RV4688				0.25		0.25							0.25			0.25				
CA_05_81_078							0.25		0.25			0.25							0.25	
CA_05_AM2003_0084							0.25		0.25			0.25							0.25	
CA_05_AM2004_0006							0.25		0.25			0.25							0.25	
CA_05_AM2004_0007							0.25		0.25			0.25							0.25	
CA_05_AM2004_0008							0.25		0.25			0.25							0.25	
CA_06_AM2004_0022								0.50												0.50
CA_06_b30071_4								0.50												0.50
CA_06_IHEM20462								0.50												0.50
CA_06_IHEM20488		0.33								0.33	0.33									
CA_06_T50								0.50												0.50
CA_07_73_024							0.25		0.25			0.25							0.25	
CA_07_b31331_6							0.25		0.25			0.25							0.25	
CA_07_HUN122				0.25		0.25							0.25			0.25				
CA_07_L1123							0.25		0.25			0.25							0.25	
CA_07_T125							0.25		0.25			0.25							0.25	
CA_08_AM2003_0059		0.33								0.33	0.33									
CA_08_b30956_5		0.33								0.33	0.33									
CA_08_HUN93		0.33								0.33	0.33									
CA_08_IHEM17983		0.33								0.33	0.33									
CA_08_YsU123		0.33								0.33	0.33									
CA_09_81_191		0.33								0.33	0.33									
CA_09_BougnCP06		0.33								0.33	0.33									
CA_09_IHEM20440		0.33								0.33	0.33									
CA_09_J981326		0.33								0.33	0.33									
CA_09_OTG10		0.33								0.33	0.33									
CA_10_Bougn17							0.25		0.25			0.25							0.25	
CA_10_Bougn27							0.25		0.25			0.25							0.25	
CO_10_R111							0.25		0.25			0.25							0.25	
CO_10_R425							0.25		0.25			0.25							0.25	
CA_11_564				0.25		0.25							0.25			0.25				
CA_11_588				0.25		0.25							0.25			0.25				
CA_11_754				0.25		0.25							0.25			0.25				
CA_11_891				0.25		0.25							0.25			0.25				
CA_11_1370				0.25		0.25							0.25			0.25				
CA_12_217														1.00						
CA_12_264														1.00						
CA_12_292														1.00						
CA_12_859														1.00						
CA_12_IHEM20415														1.00						
C. africana AM2003															1.00					
C. africana MYA2669															1.00					
CA_13_182															1.00					
CA_13_782															1.00					
CA_14_670				0.25		0.25							0.25			0.25				
CA_14_711					0.50												0.50			
CA_14_1793				0.25		0.25							0.25			0.25				
CA_14_1968				0.25		0.25							0.25			0.25				
CA_14_1969				0.25		0.25							0.25			0.25				
CA_15_172					0.50												0.50			
CA_15_191					0.50												0.50			
CA_15_643					0.50												0.50			
CA_15_759				0.25		0.25							0.25			0.25				
CA_15_928				0.25		0.25							0.25			0.25				
CA_16_357																		1.00		
CA_16_452																		1.00		
CA_16_669																		1.00		
CA_16_676				0.25		0.25							0.25			0.25				
CA_16_1397																		1.00		
CA_17_305							0.25		0.25			0.25							0.25	
CA_17_687							0.25		0.25			0.25							0.25	
CA_17_827							0.25		0.25			0.25							0.25	
CA_17_896							0.25		0.25			0.25							0.25	
CA_17_2013							0.25		0.25			0.25							0.25	
CA_18_463														1.00						
CA_18_822								0.50												0.50
CA_18_1599								0.50												0.50
CA_18_1792								0.50												0.50
CA_18_2004							0.25		0.25			0.25							0.25	

Supplementary Table E

Isolate's membership to *C. albicans* clades based on gene SYA1

	CD	AC	C1	C2	C3	C4	C5	C6	C7	C8	C9	C10	C11	C12	AF/13	C14	C15	C16	C17	C18
***C. dubliniensis* CD36**	1.00																			
**CO_AC_B41**			0.50												0.50					
**CO_AC_B60**		1.00																		
**CO_AC_B77**			0.50												0.50					
**CO_AC_R282**		1.00																		
**CO_AC_R41**		1.00																		
**CO_AC_R6**		1.00																		
CA_01_AM2003_0046			0.50												0.50					
CA_01_BougnCP01			0.50												0.50					
CA_01_SC5314			0.50												0.50					
**CO_01_B44**			0.50												0.50					
**CO_01_B80**			0.50												0.50					
CA_02_85_007				1.00																
CA_02_AM2003_0053										1.00										
CA_02_ATCC10231				1.00																
CA_02_FC13				1.00																
CA_02_T30				1.00																
CA_03_81_174					0.33	0.33	0.33													
CA_03_BougnCP11					0.33	0.33	0.33													
CA_03_C82					0.33	0.33	0.33													
CA_03_J990102					0.33	0.33	0.33													
CA_03_T65					0.33	0.33	0.33													
CA_04_b30972_4					0.33	0.33	0.33													
CA_04_IHEM16731					0.33	0.33	0.33													
CA_04_J990683										1.00										
CA_04_L343					0.33	0.33	0.33													
CA_04_RV4688					0.33	0.33	0.33													
CA_05_81_078					0.33	0.33	0.33													
CA_05_AM2003_0084					0.33	0.33	0.33													
CA_05_AM2004_0006					0.33	0.33	0.33													
CA_05_AM2004_0007					0.33	0.33	0.33													
CA_05_AM2004_0008					0.33	0.33	0.33													
CA_06_AM2004_0022								0.50	0.50											
CA_06_b30071_4								0.50	0.50											
CA_06_IHEM20462								0.50	0.50											
CA_06_IHEM20488								0.50	0.50											
CA_06_T50								0.50	0.50											
CA_07_73_024								0.50	0.50											
CA_07_b31331_6								0.50	0.50											
CA_07_HUN122								0.50	0.50											
CA_07_L1123								0.50	0.50											
CA_07_T125								0.50	0.50											
CA_08_AM2003_0059										1.00										
CA_08_b30956_5										1.00										
CA_08_HUN93										1.00										
CA_08_IHEM17983										1.00										
CA_08_YsU123										1.00										
CA_09_81_191											1.00									
CA_09_BougnCP06											1.00									
CA_09_IHEM20440			0.50												0.50					
CA_09_J981326											1.00									
CA_09_OTG10											1.00									
CA_10_Bougn17												1.00								
CA_10_Bougn27												1.00								
**CO_10_R111**												1.00								
**CO_10_R425**												1.00								
CA_11_564													0.25	0.25		0.25	0.25			
CA_11_588																		0.50		0.50
CA_11_754													0.25	0.25		0.25	0.25			
CA_11_891													0.25	0.25		0.25	0.25			
CA_11_1370																		0.50		0.50
CA_12_217																			1.00	
CA_12_264													0.25	0.25		0.25	0.25			
CA_12_292													0.25	0.25		0.25	0.25			
CA_12_859													0.25	0.25		0.25	0.25			
CA_12_IHEM20415								0.50	0.50											
C. africana AM2003			0.50												0.50					
C. africana MYA2669			0.50												0.50					
CA_13_182																			1.00	
CA_13_782																			1.00	
CA_14_670													0.25	0.25		0.25	0.25			
CA_14_711													0.25	0.25		0.25	0.25			
CA_14_1793													0.25	0.25		0.25	0.25			
CA_14_1968													0.25	0.25		0.25	0.25			
CA_14_1969													0.25	0.25		0.25	0.25			
CA_15_172													0.25	0.25		0.25	0.25			
CA_15_191													0.25	0.25		0.25	0.25			
CA_15_643																		0.50		0.50
CA_15_759													0.25	0.25		0.25	0.25			
CA_15_928													0.25	0.25		0.25	0.25			
CA_16_357																		0.50		0.50
CA_16_452																		0.50		0.50
CA_16_669																		0.50		0.50
CA_16_676																		0.50		0.50
CA_16_1397																		0.50		0.50
CA_17_305																			1.00	
CA_17_687																			1.00	
CA_17_827																			1.00	
CA_17_896																			1.00	
CA_17_2013																			1.00	
CA_18_463																		0.50		0.50
CA_18_822																		0.50		0.50
CA_18_1599																		0.50		0.50
CA_18_1792																		0.50		0.50
CA_18_2004																		0.50		0.50

Supplementary Table F

Isolate's membership to *C. albicans* clades based on gene VPS13

	CD	AC	C1	C2	C3	C4	C5	C6	C7	C8	C9	C10	C11	C12	AF/13	C14	C15	C16	C17	C18
***C. dubliniensis* CD36**	1.00																			
**CO_AC_B41**		0.17			0.17		0.17										0.17	0.17	0.17	
**CO_AC_B60**		0.17			0.17		0.17										0.17	0.17	0.17	
**CO_AC_B77**		0.17			0.17		0.17										0.17	0.17	0.17	
**CO_AC_R282**		0.17			0.17		0.17										0.17	0.17	0.17	
**CO_AC_R41**		0.17			0.17		0.17										0.17	0.17	0.17	
**CO_AC_R6**		0.17			0.17		0.17										0.17	0.17	0.17	
CA_01_AM2003_0046			1.00																	
CA_01_BougnCP01			1.00																	
CA_01_SC5314						0.33				0.33						0.33				
**CO_01_B44**			1.00																	
**CO_01_B80**						0.33				0.33						0.33				
CA_02_85_007			1.00																	
CA_02_AM2003_0053				0.25									0.25		0.25					0.25
CA_02_ATCC10231				0.25									0.25		0.25					0.25
CA_02_FC13				0.25									0.25		0.25					0.25
CA_02_T30				0.25									0.25		0.25					0.25
CA_03_81_174		0.17			0.17		0.17										0.17	0.17	0.17	
CA_03_BougnCP11		0.17			0.17		0.17										0.17	0.17	0.17	
CA_03_C82		0.17			0.17		0.17										0.17	0.17	0.17	
CA_03_J990102		0.17			0.17		0.17										0.17	0.17	0.17	
CA_03_T65				0.25									0.25		0.25					0.25
CA_04_b30972_4						0.33				0.33						0.33				
CA_04_IHEM16731						0.33				0.33						0.33				
CA_04_J990683						0.33				0.33						0.33				
CA_04_L343						0.33				0.33						0.33				
CA_04_RV4688						0.33				0.33						0.33				
CA_05_81_078								0.33	0.33		0.33									
CA_05_AM2003_0084		0.17			0.17		0.17										0.17	0.17	0.17	
CA_05_AM2004_0006		0.17			0.17		0.17										0.17	0.17	0.17	
CA_05_AM2004_0007		0.17			0.17		0.17										0.17	0.17	0.17	
CA_05_AM2004_0008		0.17			0.17		0.17										0.17	0.17	0.17	
CA_06_AM2004_0022								0.33	0.33		0.33									
CA_06_b30071_4								0.33	0.33		0.33									
CA_06_IHEM20462								0.33	0.33		0.33									
CA_06_IHEM20488								0.33	0.33		0.33									
CA_06_T50								0.33	0.33		0.33									
CA_07_73_024								0.33	0.33		0.33									
CA_07_b31331_6								0.33	0.33		0.33									
CA_07_HUN122								0.33	0.33		0.33									
CA_07_L1123								0.33	0.33		0.33									
CA_07_T125						0.33				0.33						0.33				
CA_08_AM2003_0059						0.33				0.33						0.33				
CA_08_b30956_5						0.33				0.33						0.33				
CA_08_HUN93						0.33				0.33						0.33				
CA_08_IHEM17983			1.00																	
CA_08_YsU123			1.00																	
CA_09_81_191								0.33	0.33		0.33									
CA_09_BougnCP06						0.33				0.33						0.33				
CA_09_IHEM20440								0.33	0.33		0.33									
CA_09_J981326								0.33	0.33		0.33									
CA_09_OTG10								0.33	0.33		0.33									
CA_10_Bougn17												1.00								
CA_10_Bougn27												1.00								
**CO_10_R111**												1.00								
**CO_10_R425**												1.00								
CA_11_564		0.14			0.14		0.14							0.14			0.14	0.14	0.14	
CA_11_588				0.25									0.25		0.25					0.25
CA_11_754				0.25									0.25		0.25					0.25
CA_11_891		0.14			0.14		0.14							0.14			0.14	0.14	0.14	
CA_11_1370				0.25									0.25		0.25					0.25
CA_12_217		0.14			0.14		0.14							0.14			0.14	0.14	0.14	
CA_12_264		0.14			0.14		0.14							0.14			0.14	0.14	0.14	
CA_12_292								0.33	0.33		0.33									
CA_12_859		0.14			0.14		0.14							0.14			0.14	0.14	0.14	
CA_12_IHEM20415								0.33	0.33		0.33									
C. africana AM2003				0.25									0.25		0.25					0.25
C. africana MYA2669				0.25									0.25		0.25					0.25
CA_13_182				0.25									0.25		0.25					0.25
CA_13_782				0.25									0.25		0.25					0.25
CA_14_670						0.33				0.33						0.33				
CA_14_711						0.33				0.33						0.33				
CA_14_1793						0.33				0.33						0.33				
CA_14_1968						0.33				0.33						0.33				
CA_14_1969						0.33				0.33						0.33				
CA_15_172		0.14			0.14		0.14							0.14			0.14	0.14	0.14	
CA_15_191		0.14			0.14		0.14							0.14			0.14	0.14	0.14	
CA_15_643		0.14			0.14		0.14							0.14			0.14	0.14	0.14	
CA_15_759		0.14			0.14		0.14							0.14			0.14	0.14	0.14	
CA_15_928		0.14			0.14		0.14							0.14			0.14	0.14	0.14	
CA_16_357		0.14			0.14		0.14							0.14			0.14	0.14	0.14	
CA_16_452		0.14			0.14		0.14							0.14			0.14	0.14	0.14	
CA_16_669		0.14			0.14		0.14							0.14			0.14	0.14	0.14	
CA_16_676		0.14			0.14		0.14							0.14			0.14	0.14	0.14	
CA_16_1397		0.14			0.14		0.14							0.14			0.14	0.14	0.14	
CA_17_305		0.14			0.14		0.14							0.14			0.14	0.14	0.14	
CA_17_687		0.14			0.14		0.14							0.14			0.14	0.14	0.14	
CA_17_827		0.14			0.14		0.14							0.14			0.14	0.14	0.14	
CA_17_896				0.25									0.25		0.25					0.25
CA_17_2013		0.14			0.14		0.14							0.14			0.14	0.14	0.14	
CA_18_463				0.25									0.25		0.25					0.25
CA_18_822				0.25									0.25		0.25					0.25
CA_18_1599				0.25									0.25		0.25					0.25
CA_18_1792				0.25									0.25		0.25					0.25
CA_18_2004				0.25									0.25		0.25					0.25

Supplementary Table G

Isolate's membership to *C. albicans* clades based on gene ZWF1

	CD	AC	C1	C2	C3	C4	C5	C6	C7	C8	C9	C10	C11	C12	AF/13	C14	C15	C16	C17	C18
C. dubliniensis CD36	1.00																			
**CO_AC_B41**		0.50							0.50											
**CO_AC_B60**		0.50							0.50											
**CO_AC_B77**		0.50							0.50											
**CO_AC_R282**		0.50							0.50											
**CO_AC_R41**		0.50							0.50											
**CO_AC_R6**		0.50							0.50											
CA_01_AM2003_0046			0.20	0.20								0.20	0.20							0.20
CA_01_BougnCP01			0.20	0.20								0.20	0.20							0.20
CA_01_SC5314			0.20	0.20								0.20	0.20							0.20
**CO_01_B44**			0.20	0.20								0.20	0.20							0.20
**CO_01_B80**			0.20	0.20								0.20	0.20							0.20
CA_02_85_007			0.20	0.20								0.20	0.20							0.20
CA_02_AM2003_0053			0.20	0.20								0.20	0.20							0.20
CA_02_ATCC10231			0.20	0.20								0.20	0.20							0.20
CA_02_FC13			0.20	0.20								0.20	0.20							0.20
CA_02_T30			0.20	0.20								0.20	0.20							0.20
CA_03_81_174					0.20		0.20				0.20					0.20	0.20			
CA_03_BougnCP11					0.20		0.20				0.20					0.20	0.20			
CA_03_C82					0.20		0.20				0.20					0.20	0.20			
CA_03_J990102					0.20		0.20				0.20					0.20	0.20			
CA_03_T65					0.20		0.20				0.20					0.20	0.20			
CA_04_b30972_4						0.25				0.25				0.25				0.25		
CA_04_IHEM16731						0.25				0.25				0.25				0.25		
CA_04_J990683						0.25				0.25				0.25				0.25		
CA_04_L343						0.25				0.25				0.25				0.25		
CA_04_RV4688						0.25				0.25				0.25				0.25		
CA_05_81_078					0.20		0.20				0.20					0.20	0.20			
CA_05_AM2003_0084					0.20		0.20				0.20					0.20	0.20			
CA_05_AM2004_0006					0.20		0.20				0.20					0.20	0.20			
CA_05_AM2004_0007					0.20		0.20				0.20					0.20	0.20			
CA_05_AM2004_0008					0.20		0.20				0.20					0.20	0.20			
CA_06_AM2004_0022								1.00												
CA_06_b30071_4								1.00												
CA_06_IHEM20462								1.00												
CA_06_IHEM20488								1.00												
CA_06_T50															1.00					
CA_07_73_024		0.50							0.50											
CA_07_b31331_6		0.50							0.50											
CA_07_HUN122		0.50							0.50											
CA_07_L1123		0.50							0.50											
CA_07_T125		0.50							0.50											
CA_08_AM2003_0059						0.25				0.25				0.25				0.25		
CA_08_b30956_5					0.20		0.20				0.20					0.20	0.20			
CA_08_HUN93					0.20		0.20				0.20					0.20	0.20			
CA_08_IHEM17983						0.25				0.25				0.25				0.25		
CA_08_YsU123						0.25				0.25				0.25				0.25		
CA_09_81_191					0.20		0.20				0.20					0.20	0.20			
CA_09_BougnCP06					0.20		0.20				0.20					0.20	0.20			
CA_09_IHEM20440					0.20		0.20				0.20					0.20	0.20			
CA_09_J981326					0.20		0.20				0.20					0.20	0.20			
CA_09_OTG10					0.20		0.20				0.20					0.20	0.20			
CA_10_Bougn17			0.20	0.20								0.20	0.20							0.20
CA_10_Bougn27			0.20	0.20								0.20	0.20							0.20
**CO_10_R111**			0.20	0.20								0.20	0.20							0.20
**CO_10_R425**			0.20	0.20								0.20	0.20							0.20
CA_11_564			0.20	0.20								0.20	0.20							0.20
CA_11_588					0.20		0.20				0.20					0.20	0.20			
CA_11_754			0.20	0.20								0.20	0.20							0.20
CA_11_891			0.20	0.20								0.20	0.20							0.20
CA_11_1370					0.20		0.20				0.20					0.20	0.20			
CA_12_217						0.25				0.25				0.25				0.25		
CA_12_264						0.25				0.25				0.25				0.25		
CA_12_292						0.25				0.25				0.25				0.25		
CA_12_859						0.25				0.25				0.25				0.25		
CA_12_IHEM20415						0.25				0.25				0.25				0.25		
C. africana AM2003															1.00					
C. africana MYA2669															1.00					
CA_13_182															1.00					
CA_13_782															1.00					
CA_14_670					0.20		0.20				0.20					0.20	0.20			
CA_14_711						0.25				0.25				0.25				0.25		
CA_14_1793					0.20		0.20				0.20					0.20	0.20			
CA_14_1968			0.20	0.20								0.20	0.20							0.20
CA_14_1969					0.20		0.20				0.20					0.20	0.20			
CA_15_172						0.25				0.25				0.25				0.25		
CA_15_191					0.20		0.20				0.20					0.20	0.20			
CA_15_643					0.20		0.20				0.20					0.20	0.20			
CA_15_759						0.25				0.25				0.25				0.25		
CA_15_928					0.20		0.20				0.20					0.20	0.20			
CA_16_357						0.25				0.25				0.25				0.25		
CA_16_452															1.00					
CA_16_669						0.25				0.25				0.25				0.25		
CA_16_676		0.05	0.05	0.05	0.05	0.05	0.05	0.05	0.05	0.05	0.05	0.05	0.05	0.05	0.05	0.05	0.05	0.05	0.05	0.05
CA_16_1397						0.25				0.25				0.25				0.25		
CA_17_305					0.20		0.20				0.20					0.20	0.20			
CA_17_687															1.00					
CA_17_827								1.00												
CA_17_896								1.00												
CA_17_2013						0.25				0.25				0.25				0.25		
CA_18_463			0.20	0.20								0.20	0.20							0.20
CA_18_822			0.20	0.20								0.20	0.20							0.20
CA_18_1599			0.20	0.20								0.20	0.20							0.20
CA_18_1792		0.50							0.50											
CA_18_2004			0.20	0.20								0.20	0.20							0.20
